# A potent anti-dengue human antibody preferentially recognizes the conformation of E protein monomers assembled on the virus surface

**DOI:** 10.1002/emmm.201303404

**Published:** 2014-01-14

**Authors:** Guntur Fibriansah, Joanne L Tan, Scott A Smith, Adamberage R Alwis, Thiam-Seng Ng, Victor A Kostyuchenko, Kristie D Ibarra, Jiaqi Wang, Eva Harris, Aravinda Silva, James E Crowe, Shee-Mei Lok

**Affiliations:** 1 Program in Emerging Infectious Diseases, Duke–NUS Graduate Medical SchoolSingapore City, Singapore; 2 Centre for BioImaging Sciences, National University of SingaporeSingapore City, Singapore; 3 Department of Medicine, Vanderbilt UniversityNashville, TN, USA; 4 The Vanderbilt Vaccine Center, Vanderbilt UniversityNashville, TN, USA; 5 Department of Microbiology and Immunology, University of North Carolina School of MedicineChapel Hill, NC, USA; 6 Division of Infectious Diseases and Vaccinology, School of Public Health, University of CaliforniaBerkeley, CA, USA; 7 Departments of Pediatrics and Pathology, Microbiology and Immunology, Vanderbilt UniversityNashville, TN, USA

**Keywords:** cryoEM, dengue virus, human antibody, neutralization, structure

## Abstract

Dengue virus (DENV), which consists of four serotypes (DENV1-4), infects over 400 million people annually. Previous studies have indicated most human monoclonal antibodies (HMAbs) from dengue patients are cross-reactive and poorly neutralizing. Rare neutralizing HMAbs are usually serotype-specific and bind to quaternary structure-dependent epitopes. We determined the structure of DENV1 complexed with Fab fragments of a highly potent HMAb 1F4 to 6 Å resolution by cryo-EM. Although HMAb 1F4 appeared to bind to virus and not E proteins in ELISAs in the previous study, our structure showed that the epitope is located within an envelope (E) protein monomer, and not across neighboring E proteins. The Fab molecules bind to domain I (DI), and DI-DII hinge of the E protein. We also showed that HMAb 1F4 can neutralize DENV at different stages of viral entry in a cell type and receptor dependent manner. The structure reveals the mechanism by which this potent and specific antibody blocks viral infection.

**Subject Categories** Microbiology, Virology & Host Pathogen Interaction; Immunology

## Introduction

Dengue virus (DENV), a member of the family *Flaviviridae*, is transmitted to humans by *Aedes* mosquitoes. Other flaviviruses that are important human pathogens include West Nile virus (WNV), yellow fever virus, Japanese encephalitis virus, and tick-borne encephalitis virus. DENV targets susceptible populations residing in tropical and sub-tropical regions of the globe. An estimated 400 million people worldwide are infected with DENV annually, leading to approximately 100 million cases of dengue and 21 000 deaths (Thomas ' Endy, 2011; Bhatt *et al*, 2013).

DENV consists of four distinct serotypes (DENV1–4), and the amino acid sequence variation of the polyprotein between serotypes is about 25–40% (Holmes ' Twiddy, 2003; Vasilakis ' Weaver, 2008). People infected with DENV can be asymptomatic or develop symptoms that range from a mild fever to severe dengue hemorrhagic fever (DHF) and dengue shock syndrome (DSS). A dengue-naïve individual exposed to a primary infection develops long-lasting protective immunity only to the infecting serotype (Imrie *et al*, 2007). A second infection with a new serotype increases the risk of developing DHF/DSS. The presence of cross-reactive but weakly neutralizing antibodies (Abs) induced following the primary infection have been hypothesized to be a cause of DHF or DSS through a mechanism known as Ab-dependent enhancement (Halstead, 2003). Experts in the field reason that a safe and effective vaccine against DENV will likely need to be tetravalent (Raviprakash *et al*, 2008), since the induction of Abs that neutralize a single serotype by monovalent vaccines may predispose individuals to Ab-enhanced disease.

DENV is an enveloped virus, in which the nucleocapsid core is surrounded by a lipid bilayer membrane. On the lipid envelope, there are 180 copies each of membrane (M) and envelope (E) proteins (Kuhn *et al*, 2002; Zhang *et al*, 2013a). These M and E proteins are arranged with icosahedral symmetry with each asymmetric unit consisting of three pairs of M and E proteins. The three individual E proteins in an asymmetric unit each have a different local chemical environment. The 180 copies of E protein are arranged into 90 head-to-tail homodimers. Three of these homodimers lie parallel to each other forming a raft. Together, the 30 E protein rafts on the DENV surface form a herringbone pattern (Kuhn *et al*, 2002; Zhang *et al*, 2013a).

Crystal structures of the ectodomain part of the E protein showed that it consists of three distinct domains, designated DI, DII and DIII. E protein also likely exists as a head-to-tail oriented homodimer in solution (Modis *et al*, 2003, 2005; Zhang *et al*, 2004). This molecule is critical for viral entry into cells, as it mediates binding to cellular receptors and also fusion between the virus and endosomal membranes. In addition, E protein is the major target for neutralizing Abs (Roehrig, 2003).

Studies with mouse monoclonal Abs (MAbs) showed that the most potent neutralizing Abs are serotype-specific and bind to DIII (Gromowski ' Barrett, 2007; Sukupolvi-Petty *et al*, 2007; Shrestha *et al*, 2010). In contrast, a large fraction of potent neutralizing anti-DENV Abs produced in humans does not appear to bind to DIII (Wahala *et al*, 2009, 2012; Costin *et al*, 2013). These human MAbs (HMAbs) bind only to whole virus particles but not to recombinant E (rE) protein, suggesting that they recognize quaternary structure-dependent epitopes (Beltramello *et al*, 2010; De Alwis *et al*, 2012; Teoh *et al*, 2012). One such HMAb is 1F4, a DENV1-specific Abs (De Alwis *et al*, 2012). Here, we have solved the cryo-electron microscopy (cryo-EM) structure of Fab 1F4 complexed with DENV1 to 6 Å resolution. Surprisingly, unlike other HMAbs recognizing quaternary structure-dependent epitopes (Kaufmann *et al*, 2010; Teoh *et al*, 2012), 1F4 does not bind across neighboring E proteins.

## Results

### HMAb 1F4 exhibits potent neutralizing activity *in vitro* and *in vivo*, and it inhibits virus infection at different stages of viral entry depending on the cell type and receptor

**Figure 1:**
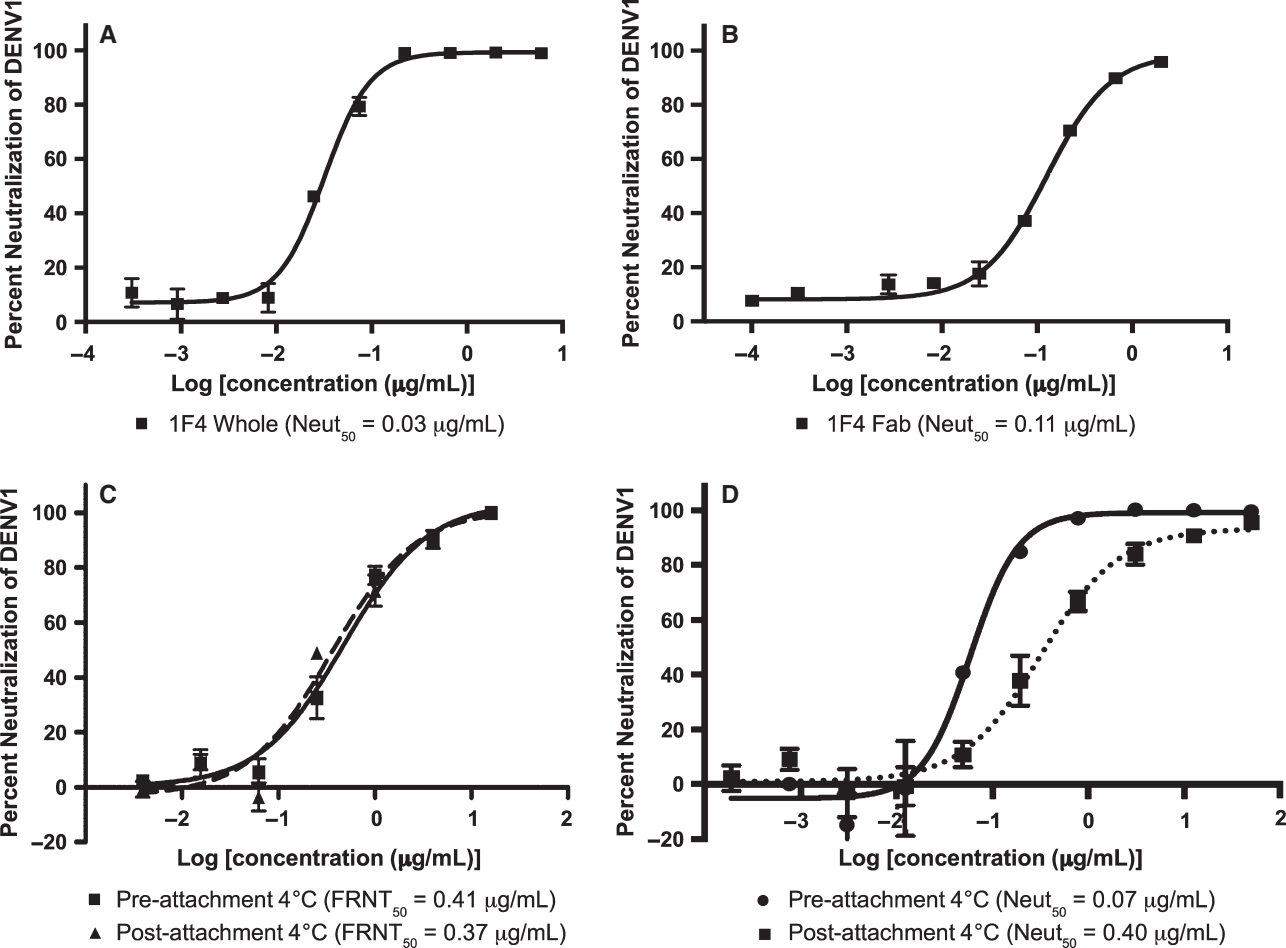
The mechanism of HMAb 1F4 neutralization of DENV1 (Western Pacific 74 strain) in Vero or DC-SIGN expressing U937 cell lines. A, B Both HMAb 1F4 IgG (A) and its Fab fragment (B) neutralized DENV1 infection in U937 cells expressing DC-SIGN although the Fab fragment required a 4-fold higher concentration. C HMAb 1F4 inhibited a post-attachment step of virus infection in Vero cells. HMAb 1F4 had similar neutralization activities when exposed to virus pre- (FRNT_50_ = 0.41 μg/ml) or post-attachment (FRNT_50_ = 0.37 μg/ml) to Vero cells. The two experiments were done with two replicates. Error bars represent standard deviations. Pre and post-attachment neutralization curves are not significantly different by a 2-way repeated measures analysis of variance (RM ANOVA). D HMAb 1F4 prevented virus infection of DC-SIGN-expressing U937 cells by blocking both virus attachment and also a post-attachment step. Error bar represented standard deviations. Neutralization curves of pre and post-attachment groups are significantly different by 2-way RM ANOVA, with a *P* < 0.001.

A neutralization assay conducted with HMAb 1F4 and DENV1 in human monocytic U937 cells expressing dendritic cell-specific intercellular adhesion molecule 3-grabbing nonintegrin (DC-SIGN) demonstrated that it is a highly potent Ab with a Neut_50_ value of 0.03 μg/ml (Fig 1A). In addition, the Fab fragment of HMAb 1F4 also neutralized the virus, although the potency of the Fab was reduced by approximately 4-fold (Neut_50_ value of 0.11 μg/ml) compared with full-length IgG (Fig 1B).

**Figure 2:**
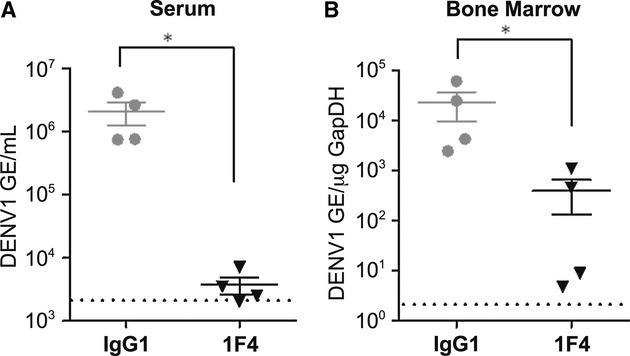
Prophylactic efficacy of HMAb 1F4 in DENV1-inoculated AG129 mice. A, B Mice were administered 20 μg of HMAb 1F4 or 50 μg IgG1 isotype control 24 h prior to a sub-lethal dose of 5 × 10^6^ pfu of DENV1 Western Pacific 74. Serum viremia (A) and bone marrow viral load (B) were determined 3 days post-infection by qRT-PCR. One representative experiment of two is shown, with *n* = 4 mice per group. DENV1 limit of detection is indicated by the dashed line. *P = 0.0209 compared to IgG1 control, as determined using a 2-tailed Wilcoxon Rank Sum test.

In mice that had been given 20 μg of HMAb 1F4 prior to infection with a sub-lethal dose of DENV1, a significant reduction in the viral genomic RNA copy number as compared to the isotype control was observed in serum and bone marrow (Fig 2A and B).

To test whether HMAb 1F4 inhibits receptor binding in Vero and DC-SIGN-expressing U937 cells, we compared the neutralization profile of HMAb 1F4 to DENV before and after the virus was allowed to attach to cells. In Vero cells, HMAb 1F4 was just as effective in neutralizing virus when the Ab was added to virus prior to exposure to cells (FRNT_50_ of 0.41 μg/ml) as when added after attachment to cells (FRNT_50_ of 0.37 μg/ml) (Fig 1C). This suggests that HMAb 1F4 neutralizes virus infection in Vero cells by inhibiting a step after virus attachment to cells. In experiments where the virus was exposed to HMAb 1F4 after attachment to cells, DC-SIGN-expressing U937 cells showed a similar neutralization profile as Vero cells (Neut_50_ of 0.40 μg/ml) (Fig 1D). However, when virus was exposed to Ab before attachment to DC-SIGN-expressing cells, the neutralizing concentration was approximately 6 fold lower (Neut_50_ of 0.07 μg/ml) (Fig 1D). These data suggest that the Ab can prevent virus from attaching to DC-SIGN-expressing cells in addition to neutralizing the virus after attachment to the cells.

### Cryo-EM structure of DENV-1 complexed with Fab 1F4

For the cryoEM reconstruction of the Fab 1F4-DENV1 complex, the DENV1 primary isolate strain PVP159 was used, and neutralization tests showed that the Ab neutralized this virus strain with high potency (supplementary Fig 1). A micrograph of an untreated control DENV1 (PVP159) (supplementary Fig 2), which was grown at 28°C in C6/36 cells and kept at 4°C, showed that the sample consisted of mainly smooth mature virus particles, with about 15% spiky immature particles and a few broken particles. DENV2 particles have been shown to exhibit a change from a smooth to a bumpy surface when incubated at 37°C for 30 min (Fibriansah *et al*, 2013; Zhang *et al*, 2013b). Incubation of DENV1 (PVP159) at 37°C for 30 min, on the other hand, did not show any increase in the number of bumpy particles compared to the sample that was never exposed to 37°C (supplementary Fig 2). This indicated that the DENV1 strain PVP159 did not undergo similar structural changes when incubated at 37°C for 30 min as had been observed in DENV2. However, we cannot eliminate the possibility of small local domain movements of the E proteins on DENV1 surface.

The expansion of the virus structure as observed in DENV2, does not seem to enhance infectivity in mammalian cells. Fibriansah *et al* (2013) showed that DENV2 titres were similar at both 37 and 28°C. This implied that both structural forms are equally infectious to mammalian cells. This indicates there may not be a strong selection pressure for the virus to adopt the expanded structure.

**Figure 3:**
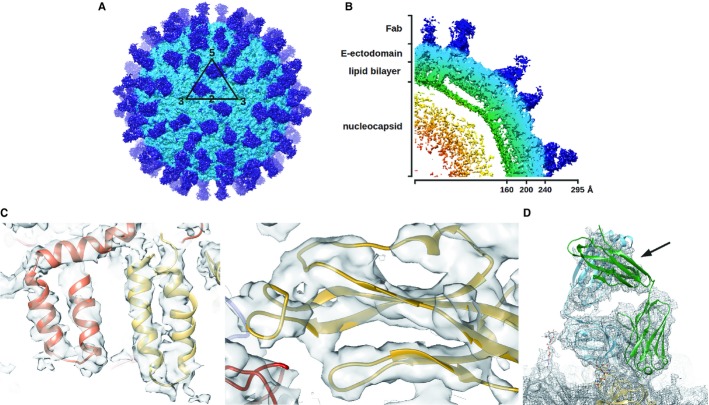
The cryo-EM structure of Fab 1F4 complexed with DENV1.
Cryo-EM map of Fab 1F4 complexed with DENV1 showed 120 copies of Fab (blue) bound to the virus surface (cyan). White triangle indicates an icosahedral asymmetric unit and the numbers represents the vertices.Cross-section of a quarter of a cryo-EM map.The resolution of the cryoEM map is 6 Å. Regions of the density map corresponding to trans-membrane α-helices (left) and β-strands (right).The density map corresponding to Fab 1F4. The density of the constant region (indicated by arrow) is much poorer than the variable region indicating the constant region is flexible.

Cryo-EM reconstruction of Fab 1F4 complexed with DENV1 strain PVP159 when incubated at 4 or 37°C resulted in similar maps. Hence, further structural analysis was done using the complex formed at 4°C as the viral components and Fab 1F4 were likely to be less mobile, thus allowing us to achieve higher resolution. The E protein shell of the cryo-EM map of the Fab-virus complex was solved to 6 Å resolution (Fig 3A–D). At this resolution, we were able to observe densities of the helical ridges (Fig 3C, left) of the E protein transmembrane region. On the other hand, the densities corresponding to the Fab molecules are poorer in resolution (Fig 3D). Resolutions of the Fab variable and constant regions were about 7.7 and 12 Å, respectively. The difference in resolution between the Fab variable and constant regions suggests high flexibility of the elbow angle between these domains (Fig 3B and D).

**Figure 4:**
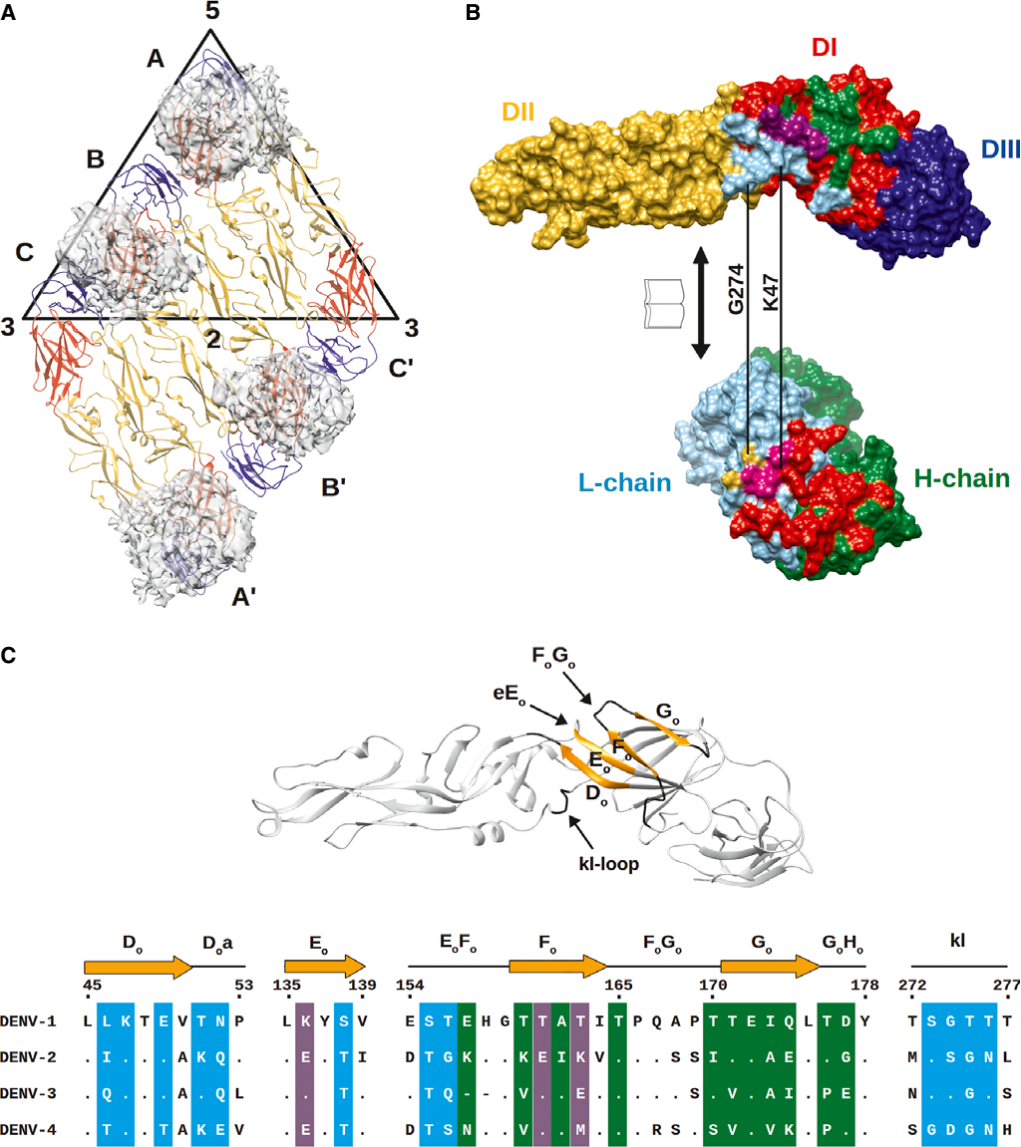
The epitope bound by Fab 1F4.
Densities of Fab 1F4 on E proteins in two icosahedral asymmetric units. Fab 1F4 molecules bind to molecules A and B in each asymmetric unit.Open-book representation of Fab 1F4 binding to E protein. The E protein DI, DII and DIII are colored in red, yellow and blue (top), respectively, whereas the Fab 1F4 heavy and light chains are colored in green and cyan (bottom), respectively. The epitope bound by Fab 1F4 is located on the DI and DI-II hinge regions (top). The epitope on E protein bound by the heavy and light chain are colored in green and cyan, respectively (top). Also the epitope on DI that interacted with both heavy and light chains are colored in purple (top). The paratope on heavy and light chain (bottom) are colored in its corresponding interacting residues on the DI (red) and DII (yellow) E protein, paratope binding to both DI and II on the E protein is colored in pink.Ribbon representation of the HMAb 1F4 epitope on E protein (top) and also sequence alignment of the epitope region between all dengue virus serotypes (bottom). The β-strand and loop of the epitope are colored in orange and black, respectively (top). In the bottom panel, the secondary structures are shown above the amino acid sequence. The amino acid residues that interact with Fab 1F4 are highlighted in the same coloring scheme as in (B). The amino acid sequences are derived from DENV1 strain PVP159, DENV2 strain S16803, DENV3 strain Thailand 1995 and DENV4 strain Dominica 1981.

**Figure 5:**
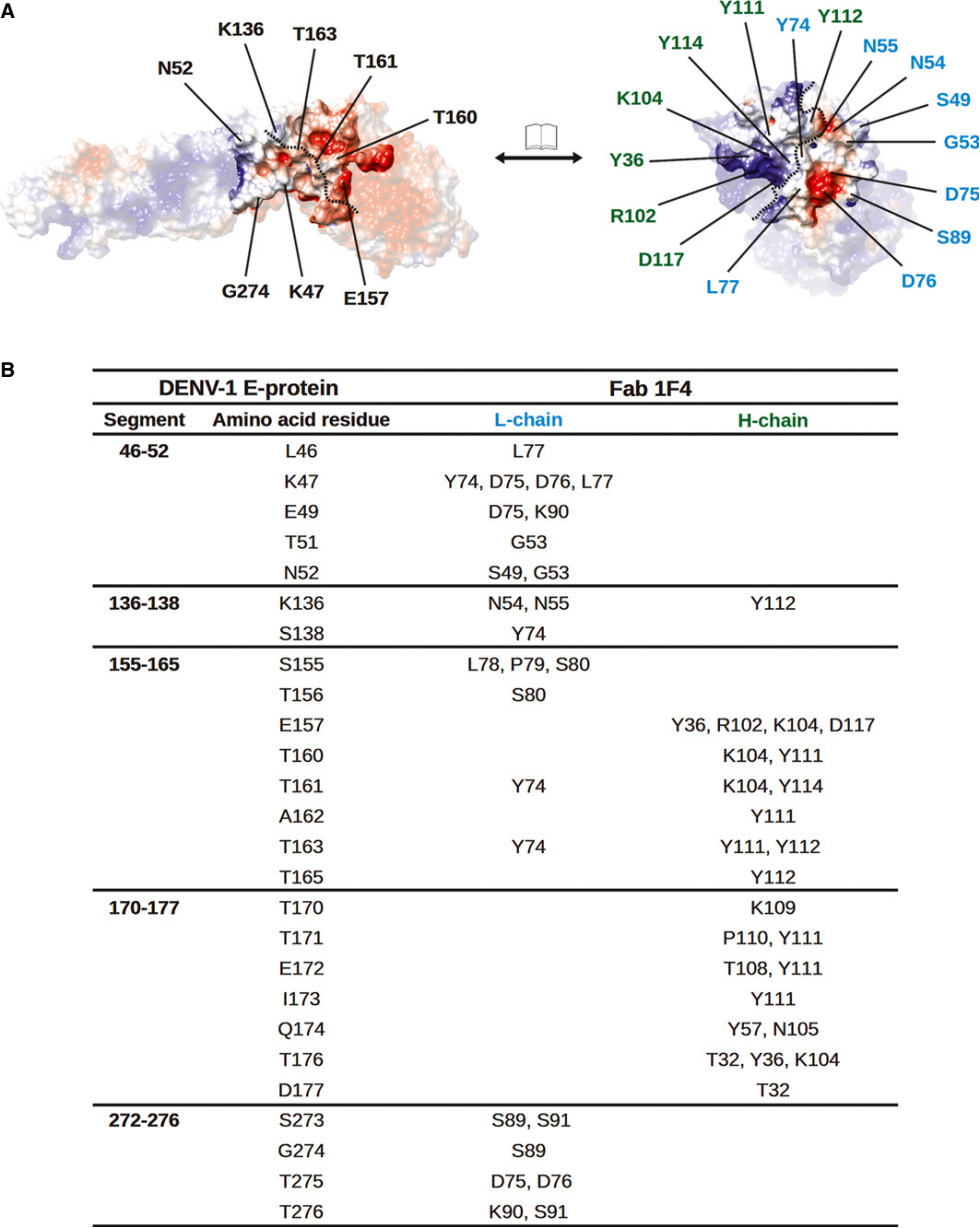
Interaction interface between Fab 1F4 and DENV1 E protein.
Open-book representation showing the electrostatic potential of the interaction interface on the E protein (left) and the Fab 1F4 (right). The blue and red colors indicate positive and negative charges, respectively, whereas the white color shows neutral charge. The dotted lines indicate the border of the footprint between heavy and light chains on the E protein epitope (left) and also the corresponding border on the antibody paratope (right). Residues on E protein identified in the neutralization escape mutants (K47 and G274) are indicated, together with other residues that have opposite charges in other serotypes. The corresponding interacting residues on the Fab 1F4 light and heavy chains are indicated with cyan and green colored fonts, respectively.Table of the list of putative interactions between DENV1 and Fab 1F4.

**Figure 6:**
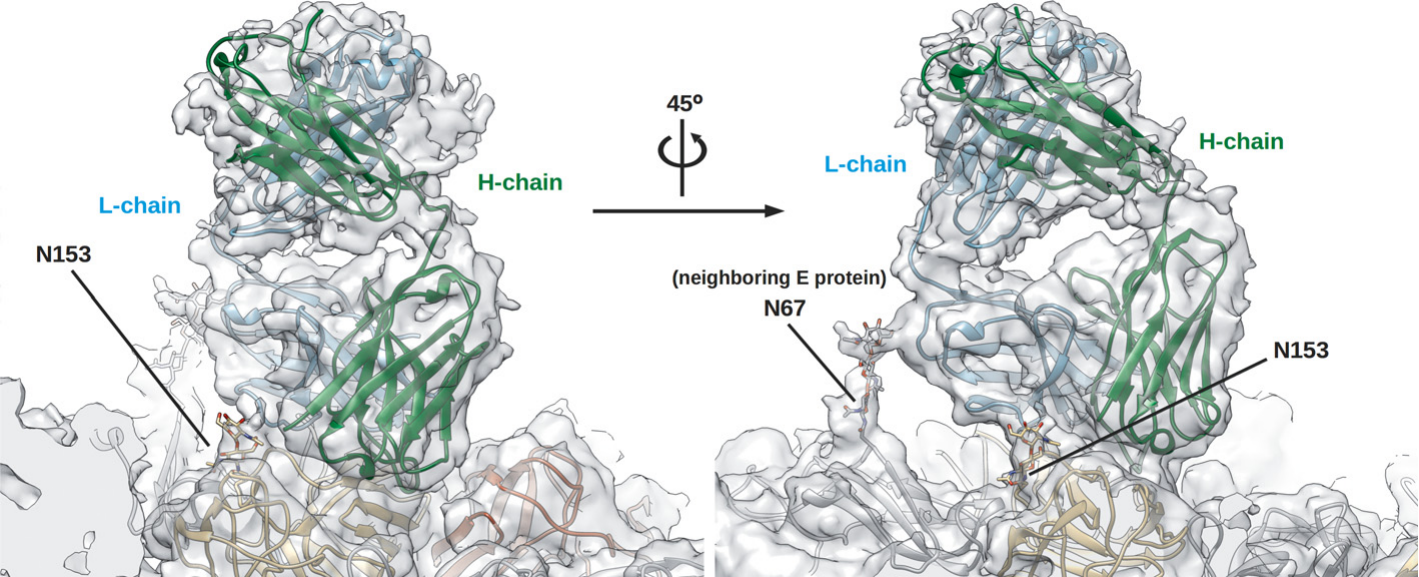
Fab 1F4 also has non-CDR related interactions with glycan chains on N153 on the same E protein and the N67 of a neighboring E protein. Left and right panels show different views of these interactions.The heavy and light chains of Fab 1F4 are colored in green and cyan, respectively, whereas the E protein molecules A and C' are in beige and gray, respectively (see Fig 4A for reference). The oxygen and nitrogen atoms of the glycan chains are colored in red and blue, respectively.

Fitting of E protein and Fab molecules into the cryo-EM density map showed that the Fab molecules bind in an identical way to two of the three individual E proteins (molecules A and B) in an asymmetric unit (Fig 4A and B). Since the resolution of the map did not permit observation of side chain densities, interacting residues between Fab and E protein were identified by observing pairs of Cα atoms of less than approximately 8 Å in distance. The possibility of hydrogen bonding and hydrophobic interactions between the side chains of these residues was also taken into consideration. The footprint of the Fab 1F4 molecule on an E protein is approximately 1340 Å^2^, which is bigger than that of a typical Ab footprint on antigen (900–1000 Å^2^) (Davies *et al*, 1990; Lok *et al*, 2008; Austin *et al*, 2012), but smaller than that of MAb E16 on WNV E protein (1550 Å^2^) (Nybakken *et al*, 2005). One Fab 1F4 molecule binds to an E protein monomer and not across E proteins. The majority of the footprint of Fab 1F4 is on E protein DI, with some interactions with the DI-DII hinge region (Fig 4B). The epitope, consisting of 26 amino acids, is located on the D_o_ strand and the downstream D_o_a loop (46–52), E_o_ strand (136–138), part of the E_o_F_o_ loop, F_o_ strand (155–165), G_o_ strand, the following G_o_H_o_ loop (170–177), and kl loop (272–276) (Fig 4C). Comparison of the epitope residues between DENV serotypes (Fig 4C) revealed significant variation, which likely explains the serotype specificity of HMAb 1F4. A total of 27 interacting residues are located on the complementarity determining regions (CDRs) of the Fab molecule: 14 residues from the light chain and 13 residues from the heavy chain (Fig 5A and B). Also, two other non-CDR related interactions were observed. The side of the Fab molecule interacted with the N153 glycosylation site on the same E protein molecule (Fig 6, left) and also the N67 glycosylation site of a neighboring dimer-related E protein (Fig 6, right).

**Figure 7:**
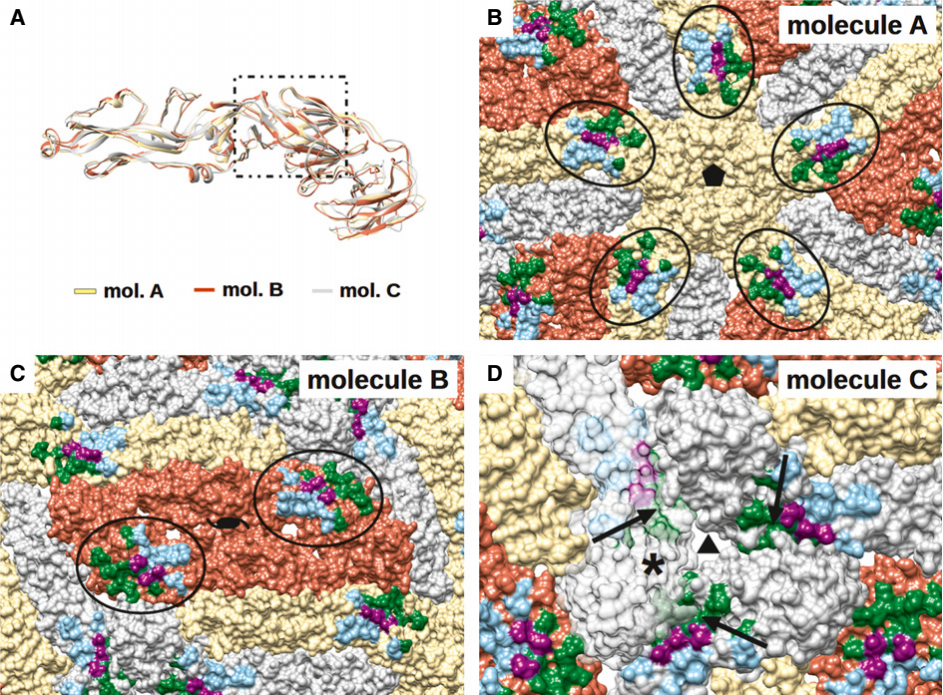
Analysis of the accessibility of the epitope on all three individual E protein molecules in an asymmetric unit. A Superposition of the three individual E protein molecules in an asymmetric unit (molecules A–C). The epitope location is indicated by a rectangular box. Molecules A, B and C in each asymmetric unit are colored in yellow, red and gray, respectively. The E proteins have similar conformation indicating that the lack of Fab binding to molecule C is mainly due to the blockage of the epitope by a neighboring E protein molecule. B–D Molecules A, B, and C in each asymmetric unit are colored in yellow, red and gray, respectively. The epitope is colored in the same coloring scheme as in Fig 4B. The epitopes on molecules A (B) and B (C) (circled) are fully accessible. (D) In contrast, the part of the epitope on molecule C is blocked by DIII of a neighboring E protein (indicated by arrow). For clarity, one of the E proteins (left) is represented as a transparent surface to show the hidden part of an adjacent epitope (marked by*).

Fab 1F4 is unable to bind to the E proteins near the 3-fold vertices (molecule C) (Fig 4A). Superposition of the three individual E protein molecules did not show significant structural differences, suggesting that the lack of binding of Fab to molecule C may be due to steric hindrance caused by the neighboring E proteins (Fig 7A). Comparison of the accessibility of the epitopes on the three E proteins in an asymmetric unit showed that the epitopes on molecules A and B are completely exposed (Fig 7B and C), while the epitope on C molecule (near to 3-fold vertices) is partially covered by DIII of a neighboring E protein (Fig 7D).

## Discussion

HMAb 1F4 is a strongly neutralizing Ab against DENV1, as shown *in vitro* (Fig 1) and in the AG129 mouse model (Fig 2). As observed previously (Beltramello *et al*, 2010; De Alwis *et al*, 2012), potent HMAbs usually bind to quaternary structure-dependent epitopes, as determined by the ability of the Ab to bind to whole virus particles and not rE protein in ELISAs. HMAb 1F4 is unique compared to the previously published HMAbs (Kaufmann *et al*, 2010; Teoh *et al*, 2012) that bind to quaternary structure-dependent epitopes, as HMAb 1F4 does not bind across the interface between separate E proteins. Instead, it recognizes E protein monomers that adopt a conformation that only exists when the protein is displayed on virus particles.

**Figure 8:**
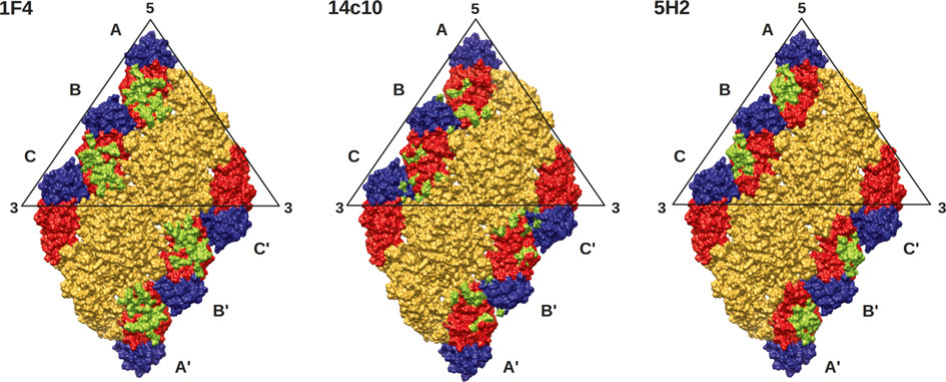
Comparison of epitopes bound by HMAb 1F4 (left), HMAb14c10 (middle) and 5H2 (right). The epitopes are colored in green.

The cryo-EM structure of another strongly neutralizing DENV1-specific HMAb, 14c10, in complex with DENV1 had been solved previously (Teoh *et al*, 2012). Comparison of the HMAb 1F4 and 14c10 epitopes showed that HMAb 1F4 binds to an E protein monomer whereas 14c10 binds across two E proteins (Fig 8). Both HMAbs 1F4 and 14c10 bind mainly to DI and the hinge between DI and DII. In addition, HMAb 14c10 also binds to the DIII of a neighboring E protein. There is another report of the crystal structure of a chimpanzee Fab 5H2 complexed with DENV4 E protein (Cockburn *et al*, 2012). The MAb 5H2 is a DENV4 specific antibody that binds to DI (Lai *et al*, 2007). The complex structure showed that the MAb 5H2 epitope is located on β-strands F_o_, G_o_,H_o_ and the loops between them as well as the loop downstream of β-strand I_o_ (DI-DIII linker). Similar interactions between Fab and amino residues on β-strands F_o_ and G_o_ and the loop F_o_G_o_ were also found in the complex structure of Fab 14c10 (Teoh *et al*, 2012) and 1F4 (Fig 4 and 8), but interactions with DI-II hinge region was not observed in the Fab 5H2 complex structure. Since the Fab 5H2 complex structure is a crystal structure, its ability to bind across E proteins and also the level of occupancy on the DENV particle surface are unknown. However, analysis of the epitopes on all three E protein molecules in an asymmetric unit suggests that the epitope would be occluded in the E protein near the three-fold vertices (molecule C) (Fig 8). The observation that the epitopes bound by HMAbs 14c10 and 1F4 overlap at the DI-DII hinge and it is not recognized by chimpanzee MAb 5H2 suggests that this region could be important in eliciting type-specific neutralizing antibody responses in humans.

**Figure 9:**
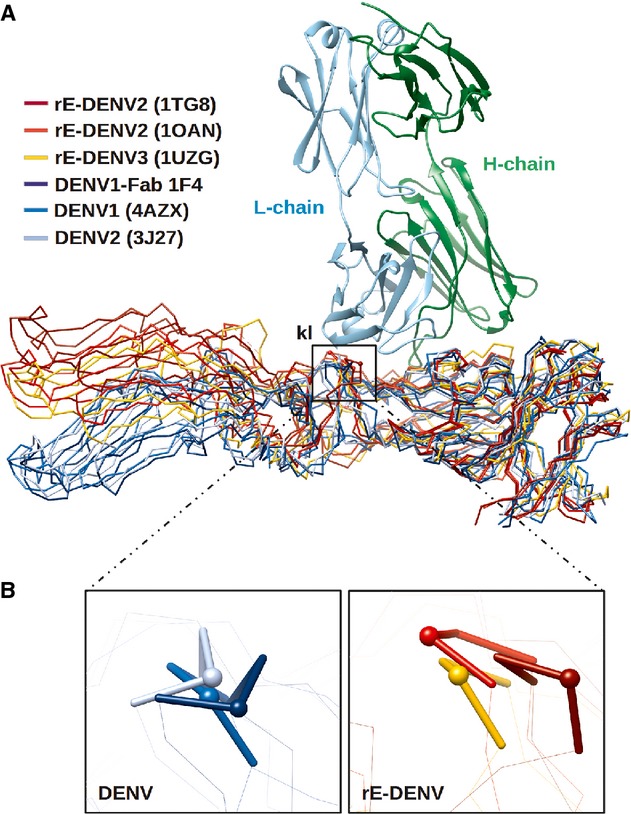
Comparison of the DI-DII hinge angle of the crystal structures of rE protein with the cryo-EM structures of virus E protein.
Superposition of the crystal structures of rE and the subnanometer resolution cryo-EM structures of virus E proteins showed that the hinge angles of rE proteins are variable whereas those on the virus surface are largely conserved. DI of these structures was superimposed using ‘LSQ superpose’ in COOT (Emsley *et al*, 2010).Zoom-in view of the part of the HMAb 1F4 epitope that consists of the kl loop (represented as sticks) in the DI-II hinge region. The kl loops from the viruses (left) showed conserved conformation whereas those from the rE proteins (right) showed varying conformations. Residue 274, which is critical for HMAb 1F4 binding (De Alwis *et al*, 2012), is shown as a sphere.

Although Fab 1F4 binds to E protein monomers on the virus, it was reported that HMAb 1F4 only binds to intact virus but not to rE protein (De Alwis *et al*, 2012). This indicates that Ab binding may requires a certain E protein conformation that is only present when the E protein is assembled on the virus. Part of the Fab 1F4 epitope is located on the kl loop (273–276) in the DI-II hinge (Fig 9A). Notably, residue 274 in the kl loop was reported to be important for HMAb 1F4 binding, since one escape mutant virus containing a substitution at this position (G274E) completely abolished Ab binding (De Alwis *et al*, 2012). When DI of the crystal structures of the DENV2 and DENV3 rE proteins were superimposed onto DI of the E proteins from the cryo-EM structures of whole virus, the conformation and orientation of the kl loop of the rE proteins were observed to be more variable than that of the virus E proteins (Fig 9A and B). The hinge angle of the E protein on the virus particle is likely to be stabilized by the interactions between E to E ectodomains and also E ectodomains with the membrane-associated E and M stem regions (Kostyuchenko *et al*, 2013) (supplementary Fig 3).

Surface electrostatic potential analysis of the interaction interface showed that the majority of interactions between Fab 1F4 and E protein on DENV are likely to be between polar residues forming hydrogen bonds or salt bridges (Fig 5A and B). The 1F4 paratope on the light (L) chain consists of a mixture of negatively charged and hydophobic residues, while those on the heavy (H) chain are largely positively charged. As expected, the H-chain contacts with the negatively charged residues of the epitope on E protein, while L-chain engages with positively charged and hydrophobic residues.

There have been two HMAb 1F4 neutralization escape mutant viruses isolated to date - one of the escape mutant viruses contained a K47E amino acid substitution and the other G274E (De Alwis *et al*, 2012). These results are consistent with the epitope identified by our cryo-EM structure. These mutations result in a change in the surface charges, likely causing the Ab to be repelled from the virus surface (Fig 5A).

Sequence comparison of the epitope residues of E proteins between DENV1-4 showed that some of the corresponding residues on DENV2-4 have opposite charges (Fig 4). For instance, in DENV1, positions 157 (Glu) and 160 (Thr) are occupied by negatively charged residues, that can interact with positive patches on the H-chain paratope (Fig 5A and B), while positively charged lysine residues are present in the same positions in DENV2. In DENV1, the lysine residue in position 136 interacts with a negatively charged patch on the border of the H- and L-chain (Fig 5A and B), while in DENV2 and DENV4, the same position contains a glutamate residue (Fig 4). Furthermore, position 274 in DENV4 consists of an aspartic acid residue. This is similar to the amino acid substitution in one of the isolated neutralization-escape mutants (G274E). A comparison between the residues in the epitope of DENV1 and DENV3 showed that there are also changes in hydrophobicity at positions L46Q, T160V, T171V, Q174I and T176P. These differences in the epitope may explain the molecular basis for the serotype specificity of the Ab.

DENV and other flaviviruses enter their host cell via receptor-mediated endocytosis (Lindenbach ' Rice, 2003; Chu ' Ng, 2004; Hidari ' Suzuki, 2011). After the virus attaches to the receptor and the complex is endocytosed, the low pH environment of the endosome causes the E proteins to rearrange from homodimers to homotrimers and in the process, expose its fusion loops. The fusion loop interacts with the endosomal membrane, which subsequently results in the fusion of the viral membrane with the endosomal membrane, thereby releasing the viral genetic material (Bressanelli *et al*, 2004; Modis *et al*, 2004). Abs can interfere with the several different steps of the infection, e.g. attachment to the receptors or ancillary receptors or E protein arrangement during fusion at low pH. By using pre- and post-attachment neutralization assays, we showed that HMAb 1F4 inhibited a post-attachment step of virus infection in Vero cells, whereas in DC-SIGN-expressing U937 cells, the Ab was also able to block virus attachment.

The previous structural study of carbohydrate recognition domains (CRD) of DC-SIGN complexed with DENV, showed that one CRD binds across two glycosylation sites at position 67 from two neighboring E proteins (molecules A and B, Fig 10) (Pokidysheva *et al*, 2006). Superposition of the cryo-EM Fab 1F4-DENV1 and the CRD-DENV complex structures suggests that HMAb 1F4 prevents attachment of the virus to DC-SIGN receptors on dendritic cells. This blocking action of the Fab is predicted to occur because when Fab 1F4 binds to virus, it will interact with the glycan at N67 on the neighboring dimer-related E protein (Fig. 6), making it unavailable for binding to the CRD. In addition, superposition of the two complexes showed a clash between Fab 1F4 and the CRD of DC-SIGN, suggesting that simultaneous binding of both molecules is not possible. This is consistent with the pre- and post-attachment neutralization tests using DC-SIGN-expressing U937 cells showing that HMAb 1F4 was able to prevent virus attachment to cells (Fig 1D).

**Figure:**
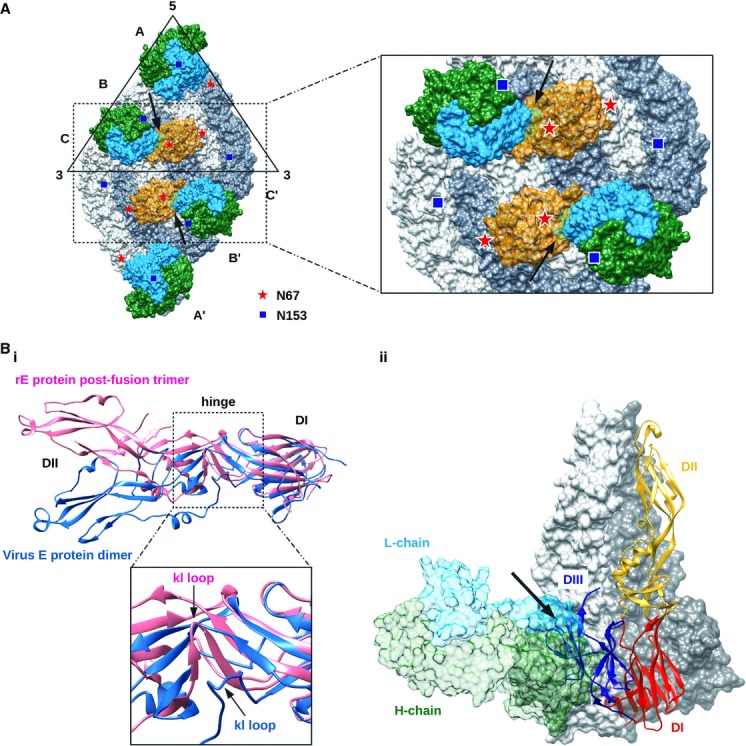
Possible neutralization mechanisms by HMAb 1F4.
HMAb 1F4 may block DC-SIGN from binding to the glycosylation site on N67. One DC-SIGN molecule binds across two N67 glycans on the virus surface. When Fab 1F4 is allowed to bind to the virus surface, it engages the N67 glycan on molecule B. This would cause steric hindrance thereby preventing the virus from binding to DC-SIGN on the surface of dendritic cells (Pokidysheva *et al*, 2006). Superposition of the cryo-EM complex structures of DENV1-Fab 1F4 and the DENV2-carbohydrate recognition domain (CRD) of DC-SIGN (PDB code 2B6B) showed that the Fab and the CRD molecules clashed. This indicates that simultaneous binding of these molecules on the virus surface is not possible. E protein molecules A, B and C are colored in light gray and molecules A’, B’ and C’ in gray. DC-SIGN is shown as a transparent orange surface. The glycosylation sites on N67 and N153 are marked as red stars and blue squares, respectively. The clashes between the Fab and DC-SIGN molecules are indicated by arrows.Fab 1F4 may interfere with the E protein dimer to trimer structural changes during fusion of the virus to the endosomal membrane. (i) The DI-DII hinge of the whole virus E protein has a different angle compared to the crystal structure of the trimeric post- fusion rE protein. This suggests that the binding of HMAb 1F4 to virus may lock the E protein hinge thus preventing the kl loop from undergoing structural changes to the post-fusion structure. The E protein on the virus and the post-fusion trimeric state are colored in blue and pink, respectively. (ii) Superposition of the cryo-EM Fab 1F4-DENV1 E protein structure onto the post-fusion trimeric E protein crystal structure. The Fab 1F4 molecule clashed with DIII of a neighboring E protein in the post-fusion trimeric structure (indicated by arrow). Two molecules of E protein are shown as surfaces (colored in light gray and dark gray) and one molecule is shown as ribbons. Fab 1F4 is drawn as a transparent surface.

The pre- and post-attachment assays using Vero or DC-SIGN-expressing U937 cells also showed that HMAb 1F4 was able to block virus infection at a post-attachment step. Flexing of the DI-DII hinge region is thought to be important for exposing the fusion loop of the virus E protein at low pH so as to facilitate the fusion of virus to the endosomal membrane (Bressanelli *et al*, 2004; Modis *et al*, 2004). HMAb 1F4, which binds to this region, may prevent the structural changes required for fusion. Superposition of the DI of E proteins of the HMAb 1F4-E complex structure with the crystal structure of the E protein post-fusion structure [PDB ID 3G7T, (Nayak *et al*, 2009)] showed that their hinge kl loop had a different conformation (Fig 10B(i)). This suggests that Ab binding to E protein on virus may lock the kl loop, preventing it from undergoing the structural changes required for fusion. Furthermore, superposition of the Fab 1F4-E protein complex and the post-fusion trimeric E protein crystal structure showed significant clashes of the Fab molecule with the neighboring E monomers in the post-fusion structure (Fig 10B(ii)). This indicates that when Fab 1F4 is bound to the virus, the E protein is not able to arrange into the post-fusion trimeric structure.

In summary, HMAb 1F4 binds to the DI and DI-II hinge regions of the E protein monomer and that Ab binding is likely to be sensitive to the hinge angle between DI-II. The overlap between the epitopes of 1F4 and another anti-DENV1 Ab 14c10, suggests that the DI-DII hinge region of the E protein is likely to be one of the principal epitopes in E protein that elicits a type-specific neutralizing Ab response. This work also suggests that the induction of neutralizing antibodies by vaccines incorporating soluble E protein monomers or dimers may be improved if the hinge angle of the E protein is identical to that on virus surface.

## Materials and Methods

### Virus sample preparation

DENV1 strains Western Pacific 74 and PVP159 were used in the present study. DENV1 strain PVP159 (DEN1/SG/07K3640DK1/2008) was obtained from the EDEN patient cohort (Low *et al*, 2006). DENV1 strains Western Pacific 74 and PVP159 were used in neutralization assays, whereas strain PVP159 was used in ELISA and the cryo-EM structure determination. DENV-1 strain PVP159 contains 14 amino acid residues changes (positions 7, 17–19, 88, 155, 161, 171, 203, 324, 339, 380, 436, and 461) in the E protein when compared to the Western Pacific 74 strain. Three of these amino acid residues are located in the epitope of HMAb 1F4, previously identified by neutralization escape mutants (De Alwis *et al*, 2012), but the amino acids are similar in properties. Specifically, PVP159 contains residues S155, T161 and T171 while the Western Pacific 74 strain contains T155, I161 and S171. Only the amino acid residue in position 161 was not replaced with a similar amino acid. However, this residue is unlikely to be important for HMAb 1F4 binding, as the Ab is able to neutralize both DENV1 strains. All viruses were grown in C6/36 *Aedes albopictus* mosquito cells at 28°C and purified as described previously (Kuhn *et al*, 2002). The purified virus was concentrated and buffer exchanged into NTE buffer (12 mM Tris–HCl pH 8.0, 120 mM NaCl and 1 mM EDTA) using an Amicon Ultra-4 centrifugal concentrator (Millipore, Billerica, MA, USA) with a 100-kDa molecular-mass cut-off filter. The purity of the virus preparation was assessed by Coomasie blue-stained SDS-PAGE. In addition to the E protein, capsid and M protein bands, it also showed a faint band at 25 kDa, which corresponded to pre-membrane (prM) protein. This indicated that there was only a slight contamination with immature virus particles. The E protein concentration was estimated by comparing the E protein band with a bovine serum albumin protein standard of known concentration.

### DENV1 neutralization assays

The anti-DENV neutralizing properties of full-length IgG or Fab fragment of HMAb 1F4 were assessed using a flow cytometry-based neutralization assay with U937 cells stably transfected with DC-SIGN as previously described (Kraus *et al*, 2007). Briefly, IgG or Fab 1F4 were serially diluted three-fold from 39 to 0.002 nM, and incubated for 1 h at 37°C with DENV1 (Western Pacific 74). The Ab-virus mix was then added to U937 cells expressing DC-SIGN and incubated at 37°C for 2 h, after which cells were washed twice with fresh infection medium and placed back at 37°C. Approximately 24 h post-infection, the cells were fixed, stained and the percentage of infected cells was assessed by flow-cytometry.

### Pre- and post-attachment neutralization assays

Pre-attachment: Vero cells in 24-well plates and reagents were cooled to 4°C. Virus and diluted Ab were incubated at 4°C for 1 h, then transferred to chilled Vero cells and incubated for another 1 h at 4°C. After incubation, cell monolayers were washed 3× with cold PBS, and cells were incubated at 37°C for 4 days before fixing and staining for foci. Pre-attachment assays with DC-SIGN expressing U937 cells were conducted similarly, with each experimental well containing 5 × 10^4^ pre-chilled cells.

Post-attachment: Vero cells in 24-well plates and reagents were chilled at 4°C. Virus was added to cells and allowed to bind for 1 h at 4°C. Unbound virus was washed off by washing twice with cold PBS. Ab was then added to virus-bound cells and incubated for 1 h at 4°C. Following incubation, cell monolayers were washed once with cold PBS and placed at 37°C for 4 days before fixing and staining for virus foci. Post-attachment neutralization assays with DC-SIGN expressing U937 cells were conducted similarly to the assay described above for Vero cells. After 24 h of infection, DC-SIGN expressing U937 cells were fixed and stained using a similar protocol described for DENV1 neutralization assay.

### AG129 mouse infections

The animal study was carried out in strict accordance with the recommendations in the Guide for the Care and Use of Laboratory Animals of the National Institutes of Health. The protocol was approved by the Animal Care and Use Committee at the University of California, Berkeley (R252-1013B). AG129 mice (van den Broek *et al*, 1995) were bred at UC Berkeley. Mice 6–8 weeks of age were administered 20 μg of hMAb 1F4 or 50 μg of an isotype control (IgG1) intraperitoneally (i.p.) in a total volume of 200 μl, 24 h prior to DENV infection. A sublethal dose of DENV1 Western Pacific 74 (5 × 10^6^ pfu) was administered intravenously (i.v.) in a total volume of 100 μl. Three days post-infection, mice were sacrificed. Serum was obtained from whole blood by centrifugation and stored at −80°C. Bone marrow cells were processed as previously described (Balsitis *et al*, 2010) by perfusing femurs with 1 ml of cold, complete RPMI containing 5% FBS, 10 mM Hepes, and 100 U penicillin/100 μg streptomycin. Processed bone marrow cells were stored short-term in RNALater (Ambion, Austin, TX, USA) at 4°C prior to RNA extraction.

### RNA extraction and quantitative RT-PCR

RNA was extracted from 20 μl of serum using the QIAamp Viral RNA mini kit (Qiagen, Valencia, CA, USA), as per the manufacturer's instructions. Similarly, RNA was extracted from the 1 ml bone marrow collection using an RNeasy Mini kit (Qiagen). The ABI Prism Sequence Detection System 7300 was used to perform quantitative RT-PCR (qRT-PCR). Viral load was determined using previously published primers and probe sequences (Johnson *et al*, 2005) and the Verso 1-Step qRT-PCR kit as follows: 2 μl RNA sample, 1X 1-step QPCR Mix, 1 μM forward and reverse DENV1 primers, 0.1 μM DENV1 probe, and Verso enzyme mix (final 1X reaction volume). The cycling parameters were as follows: 1 cycle of reverse transcription (30 min at 50°C), 1 cycle of Thermo-Start activation (12.5 min at 95°C), and 40 cycles of denaturation (15 s at 95°C) and annealing/extension (1 min at 60°C). Serum viral load was determined according to the following equation: GE/ml = (mean quantity/2 μl) (RNA extraction eluation volume/volume of serum per RNA extraction) (1000 μl/1 ml). Each DENV1 plate was run using a 10-point standard curve. To normalize for variance between tissues, bone marrow samples were analyzed for levels of GAPDH (Applied Biosystems, Taqman rodent GAPDH control kit) with manufacturer-supplied primers and probe (139 nM and 200 nM final concentration, respectively) in conjunction with the Verso 1-Step qRT-PCR kit. The GAPDH 8-point standard curve was run using control RNA provided in the Taqman rodent GAPDH control kit. GAPDH μg/ml was calculated as follows: (mean GAPDH quantity/2 μl) (dilution factor) (1000 μl/1 ml). Final GE/μg was then determined by (GE/ml) divided by (GAPDH μg/ml). The limit of detection (LOD) for the serum was based on the lowest DENV1 standard detection limit, while the bone marrow LOD (GE/μg GAPDH) was determined by dividing the DENV1 LOD by the average GAPDH levels from all bone marrow samples.

### Cryo-EM image collection and processing

DENV1 was mixed with Fab 1F4 in a molar ratio of one Fab molecule to every E protein and then incubated at one of two different temperatures (4 or 37°C), for 30 min followed by another 2 h at 4°C. The corresponding controls (DENV1 without Ab at 4 or 37°C) were also included. A 2.5 μl sample was pipetted onto ultra-thin carbon-coated lacey carbon grids (Ted Pella), and then blotted with filter paper for 2 s and flash-frozen in liquid ethane using the FEI Vitrobot Mark IV plunger. The frozen grid was kept at liquid nitrogen temperature. The virus particles were imaged using a 300-kV FEI Titan Krios electron microscope at a nominal magnification of 47 000 with an electron dose of 17.5 e/Å^2^. The images were captured on a direct electron detector (Falcon, FEI), resulting in a pixel size of 1.81 Å per pixel, at an underfocus range of 1–4 μm. Images showing drift and strong astigmatism were discarded, and 752 images were selected for further processing.

### Cryo-EM image reconstruction

Reconstruction of the DENV1-Fab complex particles was initiated by manually picking the spiky-looking unbroken particles using the program e2boxer. A total of 10 270 particles were selected and the contrast transfer function parameters were determined by using e2ctf. Following the class averaging, an initial model was generated based on a set of class-averages using e2initialmodel. The programs e2boxer, e2ctf and e2initialmodel are available in EMAN2 software package (Tang *et al*, 2007). The initial model was used in the orientation search of the particles that was carried out using Multi-Path Simulated Annealing procedure (Liu *et al*, 2007). Following the orientation search, a 3D reconstruction was done with make3d from EMAN (Ludtke *et al*, 1999). The resolution of the final map was determined by plotting the Fourier shell correlation coefficient between two half datasets-reconstructed maps with a cut-off value of 0.5.

### Model fitting

Interpretation of the map was done, by fitting the map using the cryo-EM structure of the DENV2 (Protein Data Bank [PDB] accession code 3J27). As for the Fab molecules, a homology model was built for Fab 1F4 by using Swiss-model server (Arnold *et al*, 2006). Two human Ab structures (Protein Data Bank (PDB) codes 4ERS and 3G04) were used as templates for the homology modeling of Fab 1F4 heavy and light chains, respectively. After an initial manual fitting using the program Chimera, the fit was further optimized using the “Fit in Map” command in Chimera (Pettersen *et al*, 2004). The amino acid sequences of the fitted DENV2 E and membrane proteins were then mutated to DENV1 residues by using COOT (Emsley *et al*, 2010). Further optimization of the fit for the molecules was done, by using the molecular dynamics flexible fitting (MDFF) program (Trabuco *et al*, 2009) in VMD (Humphrey *et al*, 1996) and NAMD (Phillips *et al*, 2005). Symmetry restraints were applied to avoid clashes between neighboring molecules and a factor of 0.5 was used to weigh the contribution of the cryo-EM map in the overall potential energy of the molecular dynamic simulation. The simulation involved 20 000 steps of minimization followed by 100 000 steps of molecular dynamics before converging into a stable solution. The final structure was observed to be free of misfits and clashes by using the O program (Jones *et al*, 1991).

### Structural analysis

The other structural and sequence analysis were carried out using a series of computer programs: Gerstein's accessible surface calculator (Gerstein ' Richards, 2012) in StrucTools server (http://helixweb.nih.gov/structbio/basic.html) for calculating the contact area between two molecules, Multalin (Corpet, 1988) server for multiple sequence alignment, LSQ Superpose in COOT for superimposition of protein structures, PROPKA (Li *et al*, 2005) for assigning protonation states at a particular pH, and PDB2PQR server (Dolinsky *et al*, 2004) to generate input files for evaluating the electrostatic properties of biomolecular system in Adaptive Poisson-Boltzmann Solver (Baker *et al*, 2001). Chimera was used for visualization of the structures. The amino acid sequences used for comparison were DENV2 strain S16803 (GenBank accession code ADA00411.1), DENV3 strain Thailand 1995 (GenBank accession code AY676376) and DENV4 strain Dominic 1981 (GenBank accession code AAK01233.1). The E protein structures used in the structural comparison were two crystal structures of DENV2 E proteins (PDB code 1TG8 and 1OAN), a crystal structure of DENV3 E protein (PDB code 1UZG), and two cryo-EM reconstructed structures of DENV1 and DENV2 (PDB codes 4AZX and 3J27, respectively).

### Protein structure accession number

The cryo-EM map was deposited in the Electron Microscopy Database under accession number EMD-2442. The modeled E protein – Fab 1F4 complex structure was deposited in the Protein Data Bank under accession code 4C2I.

### Statistical analysis

Data in animal experiments were analyzed using the STATA12 software (StataCorp LP, College Station, TX, USA) and a two-tailed Wilcoxon rank-sum test. Analysis of attachment assays were conducted using a 2-way repeated measures analysis of variance (RM ANOVA).

**The paper explained**

**Problem**

Dengue is a tropical disease that affects hundreds of millions of people annually and poses an economic burden in many tropical countries. There are four dengue serotypes (DENV1-4). Infection with virus from one serotype in a primary infection may potentially prime the individual to develop a more severe disease when infected with virus from another DENV serotype in a secondary infection. This is likely caused in part by antibody-dependent enhancement of infection of virus into monocyte/macrophage cells via Fcγ receptors, facilitated by the binding of non-neutralizing antibodies to virus. This phenomenon complicates the development of vaccines and therapeutics. One strategy to produce a safe dengue antibody therapeutic is to identify four potently neutralizing antibodies, each specific to a DENV serotype, and to then combine them into a cocktail. We therefore conducted structural studies to understand how a potent human antibody binds and neutralizes DENV1.

**Results**

Human monoclonal antibody (HMAb) 1F4 was shown to be highly neutralizing *in vitro* and in an AG129 mouse model. We determined the structure of DENV1 complexed with Fab 1F4 to a resolution of 6 Å by using cryo-electron microscopy (cryo-EM). The structure showed that the antibody binds to domain (D) I, and the DI-DII hinge region on an envelope protein monomer. Previous studies on HMAb 1F4 had demonstrated that it only binds to intact virus and not to recombinant envelope (rE) protein. Comparison of cryo-EM structures of virus E proteins to rE crystal structures showed that the E proteins on the virus had a conserved DI-DII hinge angle, whereas the hinge angle on the rE proteins is highly variable. As the DI-DII hinge forms part of the HMAb 1F4 epitope, we propose that HMAb 1F4 may be very sensitive to the conformation of this region. We also determined the mechanisms of neutralization of HMAb 1F4 in different cell lines. In Vero cells, the antibody prevents virus infection at a post-attachment step, whereas in DC-SIGN-expressing U937 cells, the HMAb can also prevent virus attachment. Using the cryoEM structure of 1F4 complexed with DENV, we discuss how the antibody could neutralize these steps of the virus infection.

**Impact**

We have identified and characterized an antibody that could potentially be used as a DENV1 therapeutic. The results also contribute significantly to vaccine design. Firstly, by comparing the HMAb 1F4-DENV1 structure to another potent HMAb 14c10-DENV1 structure, we observed an overlap at the DI-DII hinge, suggesting that this region is likely to be one of the principal determinants in eliciting type-specific neutralizing antibodies in humans. The inclusion of this region is thus important for the development of an effective vaccine. In addition, binding of HMAb 1F4 to E protein monomer is likely to be very sensitive to the hinge conformation. This conformation is held in proper configuration on the virus by the interactions between E ectodomains and also the E ectodomains with the E and M stem region. This has important implications for the development of an effective rE protein-based vaccine.
