# Supplementary material for: A potent anti-dengue human antibody preferentially recognizes the conformation of E protein monomers assembled on the virus surface
Source: EMBO Mol Med. 2014 Jan 14;6(3):358–71. doi: 10.1002/emmm.201303404 (PMC3958310; doi:10.1002/emmm.201303404)
Supplement: Supplementary file 1 [file emmm0006-0358-sd1.pdf]

A potent anti-dengue human antibody preferentially recognizes the conformation of E protein monomers assembled on the virus surface

Guntur Fibriansah, Joanne L. Tan, Scott A. Smith, A. Ruklanthi de Alwis, Thiam-Seng Ng, Victor A. Kostyuchenko, Kristie D. Ibarra, Jiaqi Wang, Eva Harris, Aravinda de Silva, James E. Crowe, Jr. and Shee-Mei Lok

Corresponding author: Shee-Mei Lok, Duke-NUS Graduate Medical School

Review timeline:

Submission date:	19 August 2013
Editorial Decision:	23 September 2013
Revision received:	18 October 2013
Editorial Decision:	15 November 2013
Revision received:	18 November 2013
Accepted:	20 November 2013

Transaction Report:

(Note: With the exception of the correction of typographical or spelling errors that could be a source of ambiguity, letters and reports are not edited. The original formatting of letters and referee reports may not be reflected in this compilation.)

Editor: Céline Carret

1st Editorial Decision 23 September 2013

Thank you for the submission of your manuscript to EMBO Molecular Medicine. We have now heard back from the three referees whom we asked to evaluate your manuscript.

As you will see, the three referees find the study interesting and potentially important. Nevertheless, they each have raised specific issues and suggestions to improve the interpretation, impact and readability of your work.

Given these evaluations, I would like to give you the opportunity to revise your manuscript, with the understanding that the referees' concerns must be fully addressed and that acceptance of the manuscript would entail a second round of review. Please note that it is EMBO Molecular Medicine policy to allow only a single round of revision and that, as acceptance or rejection of the manuscript will depend on another round of review, your responses should be as complete as possible.

EMBO Molecular Medicine has a "scooping protection" policy, whereby similar findings that are published by others during review or revision are not a criterion for rejection. Should you decide to submit a revised version, I do ask that you get in touch after three months if you have not completed it, to update us on the status.

Please also contact us as soon as possible if similar work is published elsewhere.

If other work is published we may not be able to extend the revision period beyond three months.

I look forward to receiving your revised manuscript.

***** Reviewer's comments *****

Referee #1 (Comments on Novelty/Model System):

This is scientifically interesting work which improves our knowledge on dengue neutralizing antibodies in humans. The manuscript represents a great deal of carefully performed work. As such this study provides important information on the design of an efficient dengue vaccine.

Referee #1 (Remarks):

The submitted manuscript by Fibriansah et al. identifies the mechanisms by which a potent and specific human monoclonal antibody (mAb) could block dengue (DEN) virus infection. The authors provide data showing that dengue serotype-specific human mAb 1F4 (de Alwis et al., Proc.Natl.Acad.Sci. USA, 2012) exhibits neutralizing activity in cultured mammalian cells infected by DEN-1 virus grown in mosquito cells and protective effect in a mouse model of virus infection. This mAb could prevent DEN-1 virus from attaching to DC-SIGN-expressing macrophage-like cells in addition to neutralizing the virus after attachment to host cells such as Vero cells. Cryo-EM analysis of DEN-1 virus complexed with Fab fragment of 1F4 revealed that this antibody binds to envelope E protein monomers that adopt a conformation that only exists when E is displayed on dengue virion. In the last part of the manuscript, the authors claim that mAb 1F4 binds to DI and DI-DII regions of the monomeric form of E. The authors suggest that DI-DII hinge region should be a major antigenic domain that elicits type-specific neutralizing antibody response in human infected with DEN-1 virus. This is scientifically interesting work which improves our knowledge on the E residues that could elicit dengue serotype-specific neutralizing antibodies in humans. As such this study also provides important information on the design of an efficient dengue vaccine. While the manuscript represents a great deal of carefully performed work, further experiments are necessary to substantiate their mechanistic study on the neutralizing activity of human mAb 1F4.

Specific points:

Major points:
Figure 1. The data on infectious DEN virus is not shown so that a full assessment of mAb 1F4 on virus replication can be made. Indeed, flow cytometry-based assay alone without DEN-1 virus titer is not so informative in understanding the inhibitory potency of mAb on viral growth. What is effect of mAb 1F4 on virus progeny production in vitro ?

In Figures 1D and 11A, the authors provide data showing that mAb 1F4 may block DC-SIGN from binding to the glycosylation site on N-67 residue of E. Several reports showed that high mannose N-glycan at position N-67 on the dimer-related E protein from DEN virus grown in mosquito cells is crucial for interaction with the CRD of DC-SIGN whereas E protein which contains complex glycosylation is unable to bind DC-SIGN. Given that carbohydrate residues present on E protein are important for virus attachment on DC-SIGN-expressing U937 cells, the authors should tested the blocking action of mAb on DEN virus grown in C6/36 cells or Vero cells. This should strenghten their hypothesis that 1F4 was able prevent virus attachment to human cells bearing DC-SIGN on their surface such as myeloid dendritic cells (DCs). Because such APCs might play a key role in the pathogenesis of dengue virus infection, the neutralizing activity of human mAb 1F4 on monocytes-derived DCs DC-SIGN+ infected with DEN-1 virus grown in mosquito cells should be examined.

Minor points
Materials and Methods. Page 13, lines 9-19. The authors need to add informations on the pre- and post-attachment neutralization assays with DC-SIGN-expressing U937 cells.
Figure 9 should be stated as Supplementary Figure 3

© EMBO

Referee #2 (Comments on Novelty/Model System):

The virological experiments were entirely solid, and the cryo-EM work was excellent. Structures of virus-Ab complexes are b no means novel, but they still provide significant insight into the mechanism of Ab neutralization, and often inform the mechanism of various events in the vial lifecycle.

Referee #2 (Remarks):

The manuscript "A potent anti-dengue human antibody preferentially recognizes the conformation of E protein monomers assembled on the virus surface" by Fibriansah et al describes a 6A cryo-EM structure of DENV1 complexed with the potent neutralizing Ab HMAb 1F4 and provides supporting virological experiments. The manuscript is technically very good: the virology is solid and necessary and the cryo-EM is of very high quality. It is also clearly written, and easy to follow. Although structures of virus-Ab complexes are b no means novel, they still provide significant insight into the mechanism of Ab neutralization, and often inform the mechanism of various events in the viral lifecycle. In particular, this manuscript clears show how the Ab recognizes a conformation of the E-protein that only exists when it is assembled on the icosahedral virus surface, thus explaining why the Ab will not recognize soluble E protein o protein from lysed virus cells. Furthermore, the manuscript sheds light on the this particular Ab is able to neutralize the virus, i.e. that when Fab 6 1F4 is bound to the virus, the E protein is not able to arrange into the post-fusion trimeric structure. This work should be of great interest to research interested in vaccine design as well as to those interested in the basic science underlying various stages in the vius lifecycle. Hence, subject to a few minor english changes, the manuscript is suitable for publiucation in EMBO Molecular Medicine.

Having said that, this reviewer believes there is additional interesting and important data in this paper that warrants discussion. In particular, the observation that DENV1, in contast to DENV2, does not change its structure upon heating to 37 degrees seems to be a significant observation. In particular, it has recently been suggested that the structural changes observed upon heating DENV2 to 37 degrees are necessary for viral infection and that this is the true structure of the virus. In contrast, the present manuscript claims that DENV1 does not change structure, suggesting that it could not use the mechanism of entry proposed form 37 degree DENV2. Although it is possible infection by DENV1 and DENV2 are different, it seems unlikely. This reviewer believes the authors have missed an opportunity to discuss an interesting and significant observation. Will this be addressed in a future manuscript?
Minor english suggestions:

Page 1:
Lines 2&3 - Maps are not "solved", the sentence should read "We determined the structure of DENV1 complexed Fab fragments of a highly potent HMAb 1F4 to 6A resolution by cryo-EM".

Page 3:
Line 9 - should read "...are arranged with icosahedral symmetry..."
Line 11 - no comma necessary

Page 6:
Lines 11 & 12: - shoulde read "...resulted in similar maps" not "...showed similar maps"

Referee #3 (Comments on Novelty/Model System):

My choices above are based in the fact that little is understood today about the way the human immune system clears dengue virus. This is a very important pathogens, and no vaccines are available. The strucutre reported here therefore provides important insight.

Referee #3 (Remarks):

This manuscript reports the cryo-EM reconstruction of dengue virus serotype 1 (DENV1) particles

in complex with a human neutralizing antibody specific for dengue 1, termed 1F4. The authors show that this antibody potently inhibits dengue serotype A virus, Western Pacific 74 strain, as well as the PVP159 strain that was actually used for the reconstruction. They also present data showing that that Mab 1F4 reduces the virus titer in mice inoculated with a sub-lethal dose of DENV1. They show that the antibody binds at a hinge region between domains I and II. The virus particle has the same herringbone arrangement already shown for DENV2 particles grown in mosquito cells at 28{degree sign}C (as was DENV1 used in this study), and also for west nile virus. Interestingly, treating the DENV1 particles to 37C does not appear to disrupt the smooth herringbone patter, although this requires a more careful analysis (see below). The resolution is close to 6≈, which allow a clear visualization of the transmembrane region of the E and M proteins. The resolution of the Fab fragment is less, especially because of the variable elbow angle between variable and constant domains. The structure shows that 120 Fabs can bind to the particle instead of 180, because 1 of the three independent epitopes is occluded at the 3-fold axes of the particle.
Overall, this is an important paper and will interest a while audience, and provide important insight into the way human antibodies recognize and neutralize infectious dengue particles. This is particularly important given that no license vaccine against dengue virus is available, and a recent phase II clinical trial was not very successful. I therefore encourage publication, provided that the authors address the specific issues listed below.
1. One main point I have is that the authors claim that the Fab can only bind to assembled virions and not to recombinant E protein. Was the status of the recombinant protein verified? Is it dimeric? It is known that the dimer affinity is not very high, and in solution the equilibrium will be shifted to monomers if the concentration is not sufficiently high. The same will happen with the protein from lysed virus. Since the authors mention that they observe contacts of the antibody also to the glycan attached at position N67 of the adjacent subunit in the dimer, such contact can only take place in the context of an E dimer. In addition, the hinge angles, in the region where the antibody binds, are more constrained in the context of a dimer than in an isolated monomer, which means that the antibody would have even less affinity for a monomer. So, in order to state that the antibody only binds in the context of a virion, they should carefully assess that they really have dimers of recombinant E protein in their tests. The authors make reference to three publications in this respect (Teoh et al, 2012; de Alwis et al, 2012; Beltramello et al, 2010), but reading them carefully, there is no indication there that the recombinant E protein used was under conditions in which there is enough dimer to detect binding. Interestingly, in page 5 (lines 20-23) of this manuscript, the authors mention that they did detect binding of Mab 1F4 at high E protein concentrations, which are conditions that induce dimerization. So, this issue of binding only on dengue virus particles should be revisited.
2 - There is another publication of a higher primate antibody bound to domain I in protein E, but in the context of dengue 4 (Cockburn et al, EMBO J., 2012), but this paper is not mentioned in the current manuscript. It would be nice to discuss a comparison of the distribution of Fab fragments in the herringbone particle seen here, with the one postulated there, in which the authors predicted that only 120 Fab molecules could bind per particle, since the epitope by the 3-fold axes was occluded. This is exactly what this paper shows happens with 1F4 on the DENV1 particle, so the comparison is relevant.
3 - Figure S2 shows that the DENV1 particles remain smooth after 30 min treatment at 37 degrees, and do not undergo the expansion shown for DENV2 under the same conditions. Although it is clear that they do not expand, it is not clear to me that they can say that they remain the same, and that some type of disorder is not introduced into the particles by this treatment. But to know that, the only way would be to make separate reconstructions with bot sets of unbound particles, and see to what resolution the herringbone pattern is maintained. Without this, the authors should be more cautious in stating that there are no changes.
Minor issues:
4. Page2, lines 20/21: The authors are most probably talking about % amino acid differences. The sequence identity between polyproteins from different serotypes is between 68% (for the most distant ones, like DEN1 and DEN4) to 78% (for the closest ones, DEN3 and DEN1). Also, the term amino acid sequence "homology" is not meaningful, since two proteins are either homologous (which is they derive from the same ancestral gene) or they are not. So it is proper to talk about % amino acid sequence identity or similarity, but not "% homology", a mistake that is frequently found. This should be corrected.

1st Revision - authors' response 18 October 2013

***** Reviewer's comments *****

Referee #1 (Comments on Novelty/Model System):

This is scientifically interesting work which improves our knowledge on dengue neutralizing antibodies in humans. The manuscript represents a great deal of carefully performed work. As such this study provides important information on the design of an efficient dengue vaccine.

Referee #1 (Remarks):

The submitted manuscript by Fibriansah et al. identifies the mechanisms by which a potent and specific human monoclonal antibody (mAb) could block dengue (DEN) virus infection. The authors provide data showing that dengue serotype-specific human mAb 1F4 (de Alwis et al., Proc.Natl.Acad.Sci. USA, 2012) exhibits neutralizing activity in cultured mammalian cells infected by DEN-1 virus grown in mosquito cells and protective effect in a mouse model of virus infection. This mAb could prevent DEN-1 virus from attaching to DC-SIGN-expressing macrophage-like cells in addition to neutralizing the virus after attachment to host cells such as Vero cells. Cryo-EM analysis of DEN-1 virus complexed with Fab fragment of 1F4 revealed that this antibody binds to envelope E protein monomers that adopt a conformation that only exists when E is displayed on dengue virion. In the last part of the manuscript, the authors claim that mAb 1F4 binds to DI and DI-DII regions of the monomeric form of E.

The authors suggest that DI-DII hinge region should be a major antigenic domain that elicits type-specific neutralizing antibody response in human infected with DEN-1 virus. This is scientifically interesting work which improves our knowledge on the E residues that could elicit dengue serotype-specific neutralizing antibodies in humans. As such this study also provides important information on the design of an efficient dengue vaccine. While the manuscript represents a great deal of carefully performed work, further experiments are necessary to substantiate their mechanistic study on the neutralizing activity of human mAb 1F4.

Specific points:

Major points:

# Figure 1. The data on infectious DEN virus is not shown so that a full assessment of mAb 1F4 on virus replication can be made. Indeed, flow cytometry-based assay alone without DEN-1 virus titer is not so informative in understanding the inhibitory potency of mAb on viral growth. What is effect of mAb 1F4 on virus progeny production in vitro?

We have done a normal PRNT in BHK cells as shown in the supplemental Figure S1, whereby the plaque was allowed to develop over 5 days. We have modified the supplementary Figure S1 to clarify that.

Change to "Figure S1. Neutralization activity of HMAb 1F4 on DENV1 (PVP159).

Both HMAb 1F4 and its Fab fragment neutralize DENV1 although the Fab fragment requires 10-fold higher concentration. This neutralization profile is similar to when DENV 1 strain West Pac was used (Fig 1). In this assay, the virus-antibody or Fab complex was added to BHK cells and the supernatant removed after 1 h incubation. RPMI/1% aquacide were layered onto the infected cells and it was then further incubated for 5 days at 37°C before staining for plaques."

The results from the $PRNT_{50}$ value using BHK cells is 0.05 µg/ml, whereas the $Neut_{50}$ value by FACS using DC-SIGN transfected U937 cells is 0.03 µg/ml. The results are similar between these assays.

# In Figures 1D and 11A, the authors provide data showing that mAb 1F4 may block DC-SIGN from binding to the glycosylation site on N-67 residue of E. Several reports showed that high mannose N-glycan at position N-67 on the dimer-related E protein from DEN virus grown in mosquito cells is crucial for interaction with the CRD of DC-SIGN whereas E protein which contains complex glycosylation is unable to bind DC-SIGN. Given that carbohydrate residues present on E protein are important for virus attachment on DC-SIGN-expressing U937 cells, the authors should tested the blocking action of mAb on DEN virus grown in C6/36 cells or Vero cells. This should strenghten their hypothesis that 1F4 was able prevent virus attachment to human cells bearing DC-SIGN on their surface such as myeloid dendritic cells (DCs). Because such APCs might play a key role in the pathogenesis of dengue virus infection, the neutralizing activity of human mAb 1F4 on monocytes-derived DCs DC-SIGN+ infected with DEN-1 virus grown in mosquito cells should be examined.

It has been previously showed that both C6/36 and Vero derived viruses have high mannose sugars because of inefficient processing in both cell lines (Hacker et al, 2009). Nevertheless, viruses derived from both cell types infected via DC-SIGN with equal efficiency. Furthermore, the fact that the maturation state of virions produced in these cells is different further complicates interpretation of results. The suggestion to repeat the neutralization studies with primary human DCs is interesting but we feel beyond the scope of this paper. We are not currently set up to make these primary human cells. This will all take time.

Hacker K, White L, de Silva AM (2009) N-linked glycans on dengue viruses grown in mammalian and insect cells. J Gen Virol 90:2097-2106.

Minor points

# Materials and Methods. Page 13, lines 9-19. The authors need to add informations on the pre- and post-attachment neutralization assays with DC-SIGN-expressing U937 cells.

The experiment details have been added to Materials and Methods, under section " Pre- and post-attachment neutralization assays" (Page 14, line 25 - Page 15, line 14)

"Pre- and post-attachment neutralization assays

Pre-attachment: Vero cells in 24-well plates and reagents were cooled to 4°C. Virus and diluted Ab were incubated at 4°C for 1 h, then transferred to chilled Vero cells and incubated for another 1 h at 4°C. After incubation, cell monolayers were washed 3x with cold PBS, and cells were incubated at 37°C for 4 days before fixing and staining for foci. Pre-attachment assays with DC-SIGN expressing U937 cells were conducted similarly, with each experimental well containing $5x10^4$ pre-chilled cells.

Post-attachment: Vero cells in 24-well plates and reagents were chilled at 4°C. Virus was added to cells and allowed to bind for 1 h at 4°C. Unbound virus was washed off by washing twice with cold PBS. Ab was then added to virus-bound cells and incubated for 1 h at 4°C. Following incubation, cell monolayers were washed once with cold PBS and placed at 37°C for 4 days before fixing and staining for virus foci. Post-attachment neutralization assays with DC-SIGN expressing U937 cells were conducted similarly to the assay described above for Vero cells. After 24 h of infection, DC-SIGN expressing U937 cells were fixed and stained using a similar protocol described for DENV1 neutralization assay."

# Figure 9 should be stated as Supplementary Figure 3

We think this figure comparing the epitope of 1F4 to the previously identified human antibodies should be shown in the main text.

Referee #2 (Comments on Novelty/Model System):

The virological experiments were entirely solid, and the cryo-EM work was excellent. Structures of virus-Ab complexes are b no means novel, but they still provide significant insight into the mechanism of Ab neutralization, and often inform the mechanism of various events in the vial lifecycle.

Referee #2 (Remarks):

The manuscript "A potent anti-dengue human antibody preferentially recognizes the conformation of E protein monomers assembled on the virus surface" by Fibriansah et al describes a 6A cryo-EM structure of DENV1 complexed with the potent neutralizing Ab HMAb 1F4 and provides supporting virological experiments. The manuscript is technically very good: the virology is solid and necessary and the cryo-EM is of very high quality. It is also clearly written, and easy to follow. Although structures of virus-Ab complexes are no means novel, they still provide significant insight into the mechanism of Ab neutralization, and often inform the mechanism of various events in the viral lifecycle. In particular, this manuscript clears show how the Ab recognizes a conformation of the E-protein that only exists when it is assembled on the icosahedral virus surface, thus explaining why the Ab will not recognize soluble E protein or protein from lysed virus cells. Furthermore, the manuscript sheds light on the this particular Ab is able to neutralize the virus, i.e. that when Fab 6 1F4 is bound to the virus, then E protein is not able to arrange into the post-fusion trimeric structure. This work should be of great interest to research interested in vaccine design as well as to those interested in the basic science underlying various stages in the vius lifecycle. Hence, subject to a few minor english changes, the manuscript is suitable for publiucation in EMBO Molecular Medicine.

Having said that, this reviewer believes there is additional interesting and important data in this paper that warrants discussion. In particular, the observation that DENV1, in contast to DENV2, does not change its structure upon heating to 37 degrees seems to be a significant observation. In particular, it has recently been suggested that the structural changes observed upon heating DENV2 to 37 degrees are necessary for viral infection and that this is the true structure of the virus. In contrast, the present manuscript claims that DENV1 does not change structure, suggesting that it could not use the mechanism of entry proposed form 37 degree DENV2.

In the paper about the warm dengue virus structure (Fibriansah et al, 2013), plaque assays were done with DENV2 incubated at 28°C and 37°C, the former will contain only smooth virus particles whereas the latter contains the expanded particles. The virus titer did not show significant differences indicating that in both structural forms they are equally infectious to mammalian cells. Whether the two structural forms enter mammalian cells in the same way is unknown. Even if they use a different mode of entry into cells, it would be an equally efficient way since the virus titer did not change.

Although it is possible infection by DENV1 and DENV2 are different, it seems unlikely.

There have been reports on dengue virus serotypes using different sets of receptors for entry (Lin et al, 2002; Thepparit and Smith, 2004).

Lin YL, Lei HY, Lin YS, Yeh TM, Chen SH, Liu HS (2002) Heparin inhibits dengue-2 virus infection of five human liver cell lines. Antiviral Res 56:93-96

Theparrit C, Smith DR (2004) Serotype-specific entry of dengue virus into liver cells: Identification of the 37-Kilodalton/67-Kilodalton high-affinity laminin receptor as a dengue virus serotype 1 receptor. J Virol 78:12647-12656.

This reviewer believes the authors have missed an opportunity to discuss an interesting and significant observation. Will this be addressed in a future manuscript?

We have now included a paragraph after the first paragraph under the header "Cryo-EM structure of DENV-1 complexed with Fab 1F4" in the results section": "The expansion of the virus structure as observed in DENV2, does not seem to enhance infectivity in mammalian cells. Fibriansah et al (2013) showed that DENV2 titres were similar at both 37°C and 28°C. This implied that both structural forms are equally infectious to mammalian cells. This indicates there may not be a strong selection pressure for the virus to adopt the expanded structure."

Minor English suggestions:

Page 1:

Lines 2&3 - Maps are not "solved", the sentence should read "We determined the structure of DENV1 complexed Fab fragments of a highly potent HMAb 1F4 to 6A resolution by cryo-EM".

Corrected.

Page 3:

Line 9 - should read "...are arranged with icosahedral symmetry..."

Line 11 - no comma necessary

Corrected

Page 6:

Lines 11 & 12: - should read "...resulted in similar maps" not "...showed similar maps"

Corrected

Referee #3 (Comments on Novelty/Model System):

My choices above are based in the fact that little is understood today about the way the human immune system clears dengue virus. This is a very important pathogens, and no vaccines are available. The structure reported here therefore provides important insight.

Referee #3 (Remarks):

This manuscript reports the cryo-EM reconstruction of dengue virus serotype 1 (DENV1) particles

in complex with a human neutralizing antibody specific for dengue 1, termed 1F4. The authors show that this antibody potently inhibits dengue serotype A virus, Western Pacific 74 strain, as well as the PVP159 strain that was actually used for the reconstruction. They also present data showing that that Mab 1F4 reduces the virus titer in mice inoculated with a sub-lethal dose of DENV1. They show that the antibody binds at a hinge region between domains I and II. The virus particle has the same herringbone arrangement already shown for DENV2 particles grown in mosquito cells at 28°C (as was DENV1 used in this study), and also for west nile virus. Interestingly, treating the DENV1 particles to 37°C does not appear to disrupt the smooth herringbone patter, although this requires a more careful analysis (see below). The resolution is close to 6Å;, which allow a clear visualization of the transmembrane region of the E and M proteins. The resolution of the Fab fragment is less, especially because of the variable elbow angle between variable and constant domains. The structure shows that 120 Fabs can bind to the particle instead of 180, because 1 of the three independent epitopes is occluded at the 3-fold axes of the particle.

Overall, this is an important paper and will interest a while audience, and provide important insight into the way human antibodies recognize and neutralize infectious dengue particles. This is particularly important given that no license vaccine against dengue virus is available, and a recent phase II clinical trial was not very successful. I therefore encourage publication, provided that the authors address the specific issues listed below.

1. One main point I have is that the authors claim that the Fab can only bind to assembled virions and not to recombinant E protein. Was the status of the recombinant protein verified? Is it dimeric? It is known that the dimer affinity is not very high, and in solution the equilibrium will be shifted to monomers if the concentration is not sufficiently high. The same will happen with the protein from lysed virus. Since the authors mention that they observe contacts of the antibody also to the glycan attached at position N67 of the adjacent subunit in the dimer, such contact can only take place in the context of an E dimer. In addition, the hinge angles, in the region where the antibody binds, are more constrained in the context of a dimer than in an isolated monomer, which means that the antibody would have even less affinity for a monomer. So, in order to state that the antibody only binds in the context of a virion, they should carefully assess that they really have dimers of recombinant E protein in their tests.

The authors make reference to three publications in this respect (Teoh et al, 2012; de Alwis et al, 2012; Beltramello et al, 2010), but reading them carefully, there is no indication there that the recombinant E protein used was under conditions in which there is enough dimer to detect binding. Interestingly, in page 5 (lines 20-23) of this manuscript, the authors mention that they did detect binding of Mab 1F4 at high E protein concentrations, which are conditions that induce dimerization. So, this issue of binding only on dengue virus particles should be revisited.

We have conducted a non-reducing SDS-PAGE gel to analyze the oligomerization states of the E protein at different concentrations.

We have included the gel in Figure 3C and have added a section in the results.

“The oligomerization states of rE protein at 20, 100 and 500 mg/mL

In the competitive ELISA binding assay, at rE protein concentrations of 20 µg/mL, it did not inhibit 1F4 binding to virus. On the other hand, at 100 **m**g/mL and 500 **m**g/mL, it inhibits ~5% and 20% of the 1F4 binding to virus, respectively. To correlate the oligomerization status of the E proteins at these concentrations, we have analyzed E proteins in a non-reducing SDS-PAGE gel. Similar total amounts of proteins for each concentration were loaded onto the wells so as to allow quantitative comparisons (Fig 3C). The E proteins were detected by using western blots (WB) and the intensity of the bands was measured. The results showed that there is an increased in E protein oligomerization states at higher concentrations (Fig 3C). We observed bands that corresponded to E protein dimers (80kDa), trimers (120kDa) and tetramers (run at ~150kDa). At 20 **m**g/mL, the E proteins mainly exist as monomers with only 24% dimers and very low amounts of trimers and tetramers. On the other hand, at 500 mg/mL, the monomeric state was reduced to 37% while the dimeric, trimeric and tetrameric states were increased to 30%, 18% and 16%, respectively. Therefore at 500 **m**g/mL, 320 **m**g/mL of the E proteins existed as multimers (dimers, trimers and tetramers). In the competitive binding ELISAs, 10 µg/mL of 1F4 was mixed with an equal volume of 500 µg/mL rE protein (of which, 320 **m**g/mL are multimers) prior to addition to virus coated on

plates. This will equate to one 1F4 Fab to every 53 molecules of the multimeric E proteins. On the other hand, if we considered only the E protein dimers (due to the uncertainty of the structures of trimers and tetramers), this ratio becomes one 1F4 Fab to every 25 molecules of the dimeric E proteins. Even in this excess, the competitive ELISAs showed only 20% inhibitory effect on 1F4 binding to virus, thus suggesting that E protein oligomerization does not improve antibody binding."

We have also added a paragraph in the Discussion section, paragraph 4

"The side of the Fab 1F4 molecule (non-CDR related) was observed to interact with glycosylation sites on N153 from the same E protein and N67 from the dimer-related E protein. The rE protein also should contain these glycosylation sites, since these proteins were expressed in *Drosophila* cells. Our WB results also showed that the rE protein exist as dimers, trimers and tetramers when the concentration is increased, however, even large excess of these higher oligomerization states of the E protein could only inhibit 20% of the MAb 1F4 binding to virus as shown in the competitive binding assay (Fig 3B and C). Thus the poor binding of HMAb 1F4 to rE suggests that these glycosylation sites are not sufficient for binding."

We also have added a sub-section "Non-reducing SDS-PAGE" in Materials and Methods, after sub-section "ELISA binding assays"

"Non-reducing SDS-PAGE gel

E-protein at three different concentrations (20, 100 and 500 µg/mL) in 100 mM Tris buffer pH 8.0 were mixed with non-reducing SDS-PAGE sample loading buffer prior to loading onto a 4-15% SDS-PAGE gel (Biorad). The E protein samples were not boiled and β-mercaptoethanol was not added. About 0.5 µg proteins for each E protein concentration were loaded into the wells and the gel was run in running buffer (25 mM Tris, 192 mM glycine, 0.1 % SDS, pH ~8.3). The protein bands were transferred onto the immuno-blot PVDF membrane (Biorad) by using Trans-blot, a semi dry electrophoretic transfer cell (Biorad). The membrane was then blocked with 5% skim-milk in phosphate buffer pH 7.4. HMAb 4.8A and HRPO conjugated goat anti-human IgG (H+L) antibody (Invitrogen) were used as primary and secondary antibodies, respectively. The protein bands were detected by using the ECL plus western blotting detection system (Amersham GE Healthcare). The band intensities were quantitated by using the ImageJ, a image processing program (Schneider et al, 2012)."

2 - There is another publication of a higher primate antibody bound to domain I in protein E, but in the context of dengue 4 (Cockburn et al, EMBO J., 2012), but this paper is not mentioned in the current manuscript. It would be nice to discuss a comparison of the distribution of Fab fragments in the herringbone particle seen here, with the one postulated there, in which the authors predicted that only 120 Fab molecules could bind per particle, since the epitope by the 3-fold axes was occluded. This is exactly what this paper shows happens with 1F4 on the DENV1 particle, so the comparison is relevant.

The structure comparison with the aforementioned antibody (5H2) has been added to the Discussion section, second paragraph.

"The cryo-EM structure of another strongly neutralizing DENV1-specific HMAb, 14c10, in complex with DENV1 had been solved previously (Teoh et al, 2012). Comparison of the HMAb 1F4 and 14c10 epitopes showed that HMAb 1F4 binds to an E protein monomer whereas 14c10 binds across two E proteins (Fig 9). Both HMAbs 1F4 and 14c10 bind mainly to DI and the hinge between DI and DII. In addition, HMAb 14c10 also binds to the DIII of a neighboring E protein. There is another report of the crystal structure of a chimpanzee Fab 5H2 complexed with DENV4 E protein (Cockburn et al, 2012). The MAb 5H2 is a DENV4 specific antibody that binds to DI (Lai et al, 2007). The complex structure showed that the MAb 5H2 epitope is located on b-strands F_0, G_0,H_0 and the loops between them as well as the loop downstream of b-strand I_0 (DI-DIII linker). Similar interactions between Fab and amino residues on b-strands F_0 and G_0 and the loop F_0G_0 were also found in the complex structure of Fab 14c10 (Teoh et al, 2012) and 1F4 (Fig 5 and 9), but

interactions with DI-II hinge region was not observed in the Fab 5H2 complex structure. Since the Fab 5H2 complex structure is a crystal structure, its ability to bind across E proteins and also the level of occupancy on the DENV particle surface are unknown. However, analysis of the epitopes on all three E protein molecules in an asymmetric unit suggests that the epitope would be occluded in the E protein near the 3-fold vertices (molecule C) (Fig 9). The observation that the epitopes bound by HMAbs 14c10 and 1F4 overlap at the DI-DII hinge and it is not recognized by chimpanzee MAb 5H2 suggests that this region could be important in eliciting type-specific neutralizing antibody responses in humans."

The figure of 5H2 epitope has been included in Fig 9.

3 - Figure S2 shows that the DENV1 particles remain smooth after 30 min treatment at 37 degrees, and do not undergo the expansion shown for DENV2 under the same conditions. Although it is clear that they do not expand, it is not clear to me that they can say that they remain the same, and that some type of disorder is not introduced into the particles by this treatment. But to know that, the only way would be to make separate reconstructions with both sets of unbound particles, and see to what resolution the herringbone pattern is maintained. Without this, the authors should be more cautious in stating that there are no changes.

We changed the sentence in line 6, page 7 from:

"This indicated that the DENV1 strain PVP159 did not undergo structural changes when incubated at 37°C for 30 min as had been observed in DENV2." to

"This indicated that the DENV1 strain PVP159 did not undergo similar structural changes when incubated at 37°C for 30 min as had been observed in DENV2. However, we cannot eliminate the possibility of small local domain movements of the E proteins on DENV1 surface."

Minor issues:

4. Page2, lines 20/21: The authors are most probably talking about % amino acid differences. The sequence identity between polyproteins from different serotypes is between 68% (for the most distant ones, like DEN1 and DEN4) to 78% (for the closest ones, DEN3 and DEN1). Also, the term amino acid sequence "homology" is not meaningful, since two proteins are either homologous (which is they derive from the same ancestral gene) or they are not. So it is proper to talk about % amino acid sequence identity or similarity, but not "% homology", a mistake that is frequently found. This should be corrected.

We changed the sentence in line 20-21, page 2

from: " DENV consists of four distinct serotypes (DENV1-4), and the amino acid sequence homology of the polyprotein between serotypes is about 25-40%" to DENV consists of four distinct serotypes (DENV1-4), and the amino acid sequence variation of the polyprotein between serotypes is about 25-40%"

2nd Editorial Decision 15 November 2013

Thank you for the submission of your revised manuscript to EMBO Molecular Medicine. We have now received the enclosed reports from the referees that were asked to re-assess it. As you will see the reviewers are now globally supportive and I am pleased to inform you that we will be able to accept your manuscript pending the following final amendments:

I would like to strongly encourage you to follow referee #2 suggestion to remove figure 3 and tone down your conclusion regarding the specific binding to visions as indeed, this figure does not convincingly address this point.

Please submit your revised manuscript within two weeks. I look forward to seeing a revised form of your manuscript as soon as possible.

***** Reviewer's comments *****

Referee #1 (Remarks):

The revised version of the manuscript is now suitable for publication in EMBO Molecular Medicine journal in according to the responses and modifications that have been given by the authors

Referee #3 (Remarks):

The revised manuscript by Fibriansah et al is very much improved with respect to the previous version. The only section that is not convincing is the quality of the recombinant E protein used to test binding by the antibody, and for inhibition of virus neutralization by Mab 1F4 by competition. First, native gels are not the best approach to investigate the oligomeric status of a protein - size exclusion chromatography combined to multi angle static light scattering (known by "MALS") would be a much better approach. If anything, the experiments displayed in the new Figure 3 indicate that the quality of the recombinant E protein used is very poor, giving rise to bands in the gel that the authors interpret as trimers and tetramers, in addition to monomers and dimers, which may be the result of partial aggregation of an improperly folded protein. Even in the case of Mab 4G2, which binds to a simpler epitope, the signal appears to be poor (Fig 3A). But the fact that the authors cannot prove the status of their E protein does not change anything to the quality of their results, they only need to omit saying that the antibody binds only to virus particles, and the rest of the manuscript holds. They could say that it appears to bind only to whole virions, and not try to make a point for which they have no data.
In conclusion, if Figure 3 is removed and the authors temper their assertion that the 1F4 MAb binds exclusively to virions, I'm ready to accept this otherwise very nice manuscript.

2nd Revision - authors' response 18 November 2013

We have now removed Figure 3 and tone down the conclusion regarding the specific binding of antibody to virus.

****** Reviewer's comments ******

Referee #3 (Remarks):

The revised manuscript by Fibriansah et al is very much improved with respect to the previous version. The only section that is not convincing is the quality of the recombinant E protein used to test binding by the antibody, and for inhibition of virus neutralization by Mab 1F4 by competition. First, native gels are not the best approach to investigate the oligomeric status of a protein - size exclusion chromatography combined to multi angle static light scattering (known by "MALS") would be a much better approach. If anything, the experiments displayed in the new Figure 3 indicate that the quality of the recombinant E protein used is very poor, giving rise to bands in the gel that the authors interpret as trimers and tetramers, in addition to monomers and dimers, which may be the result of partial aggregation of an improperly folded protein. Even in the case of Mab 4G2, which binds to a simpler epitope, the signal appears to be poor (Fig 3A). But the fact that the authors cannot prove the status of their E protein does not change anything to the quality of their results, they only need to omit saying that the antibody binds only to virus particles, and the rest of the manuscript holds. They could say that it appears to bind only to whole virions, and not try to make a point for which they have no data. In conclusion, if Figure 3 is removed and the authors temper their assertion that the 1F4 MAb binds exclusively to virions, I'm ready to accept this

otherwise very nice manuscript.

We changed the abstract from:

"Dengue virus (DENV), which consists of four serotypes (DENV1-4), infects over 400 million people annually. Previous studies have indicated most human monoclonal antibodies (HMAbs) from dengue patients are cross-reactive and poorly neutralizing. Rare neutralizing HMAbs are usually serotype-specific and bind to quaternary structure-dependent epitopes. We determined the structure of DENV1 complexed with Fab fragments of a highly potent HMAb 1F4 to 6Å resolution by cryo-EM. The Fab molecules bind to domain I (DI), and DI-DII hinge on an envelope (E) protein monomer. HMAb 1F4 binds specifically to E proteins on the virus surface and not to recombinant envelope protein (rE) or the E protein from lysed virus. Comparison of the available crystal structures of rE with the cryo-EM structures of the E protein on virions suggests that HMAb 1F4 requires a specific DI-DII hinge angle for recognition. We also showed that HMAb 1F4 can neutralize DENV at different stages of viral entry in a cell type and receptor dependent manner. The structure reveals the mechanism by which this potent and specific antibody blocks viral infection."

to

"Dengue virus (DENV), which consists of four serotypes (DENV1-4), infects over 400 million people annually. Previous studies have indicated most human monoclonal antibodies (HMAbs) from dengue patients are cross-reactive and poorly neutralizing. Rare neutralizing HMAbs are usually serotype-specific and bind to quaternary structure-dependent epitopes. We determined the structure of DENV1 complexed with Fab fragments of a highly potent HMAb 1F4 to 6Å resolution by cryo-EM. Although HMAb 1F4 appeared to bind to virus and not E proteins in ELISAs in the previous study, our structure showed that the epitope is located within an envelope (E) protein monomer, and not across neighboring E proteins. The Fab molecules bind to domain I (DI), and DI-DII hinge of the E protein. We also showed that HMAb 1F4 can neutralize DENV at different stages of viral entry in a cell type and receptor dependent manner. The structure reveals the mechanism by which this potent and specific antibody blocks viral infection."

We removed the last sentence of the last paragraph of the introduction section (Page 4, lines 4-6)

"The binding of HMAb 1F4 to E protein monomers on the virus surface and not soluble rE proteins suggests that HMAb 1F4 binds specifically to a certain E protein conformation that exists only when displayed on the intact virus surface. "

We removed the Discussion part sub-sections "HMAb 1F4 binds strongly to E proteins on whole virus particles but poorly to rE soluble protein or full-length E protein from lysed virus" and "The oligomerization states of rE protein at 20, 100 and 500 mg/mL" including Figure 3 from the manuscript.

We changed paragraph 3 of Discussion section (page 10 lines 3-15) from:

"Although Fab 1F4 binds to E protein monomers on the virus, it exhibits low or no binding to rE protein (Fig 3A and B) or E proteins from lysed virus (Fig 3A) in ELISAs. This indicates that Ab binding requires a certain E protein conformation that is only present when the E protein is assembled on the virus. Part of the Fab 1F4 epitope is located on the kl loop (273-276) in the DI-II hinge (Fig 10A). Notably, residue 274 in the kl loop was reported to be important for HMAb 1F4 binding, since one escape mutant virus containing a substitution at this position (G274E) completely abolished Ab binding (de Alwis et al, 2012). When DI of the crystal structures of the DENV2 and DENV3 rE proteins were superimposed onto DI of the E proteins from the cryo-EM structures of whole virus, the conformation and orientation of the kl loop of the rE proteins were observed to be more variable than that of the virus E proteins (Fig 10A, B). The hinge angle of the E protein on the

virus particle is likely to be stabilized by the interactions between E to E ectodomains and also E ectodomains with the membrane-associated E and M stem regions (Kostyuchenko et al, 2013) (Supplementary Fig S3)."

to:

"Although Fab 1F4 binds to E protein monomers on the virus, it was reported that HMAb 1F4 only binds to intact virus but not to rE protein (de Alwis et al, 2012). This indicates that Ab binding may requires a certain E protein conformation that is only present when the E protein is assembled on the virus. Part of the Fab 1F4 epitope is located on the kl loop (273-276) in the DI-II hinge (Fig 9A). Notably, residue 274 in the kl loop was reported to be important for HMAb 1F4 binding, since one escape mutant virus containing a substitution at this position (G274E) completely abolished Ab binding (de Alwis et al, 2012). When DI of the crystal structures of the DENV2 and DENV3 rE proteins were superimposed onto DI of the E proteins from the cryo-EM structures of whole virus, the conformation and orientation of the kl loop of the rE proteins were observed to be more variable than that of the virus E proteins (Fig 9A, B). The hinge angle of the E protein on the virus particle is likely to be stabilized by the interactions between E to E ectodomains and also E ectodomains with the membrane-associated E and M stem regions (Kostyuchenko et al, 2013) (Supplementary Fig S3)."

We removed paragraph 4 of Discussion section (Page 10, lines 16-23):

"The side of the Fab 1F4 molecule (non-CDR related) was observed to interact with glycosylation sites on N153 from the same E protein and N67 from the dimer-related E protein. The rE protein also should contain these glycosylation sites, since these proteins were expressed in *Drosophila* cells. Our WB results also showed that the rE protein exist as dimers, trimers and tetramers when the concentration is increased, however, even large excess of these higher oligomerization states of the E protein could only inhibit 20% of the MAb 1F4 binding to virus as shown in the competitive binding assay (Fig 3B and C). Thus the poor binding of HMAb 1F4 to rE suggests that these glycosylation sites are not sufficient for binding."

We changed last paragraph of Discussion section (Page 13, lines 6-14) from:

"In summary, HMAb 1F4 binds to the DI and DI-II hinge regions of the E protein monomer and that Ab binding is likely to be sensitive to the hinge angle between DI-II. The structural data in this paper showed that the E protein must be in a conformation similar to that on the virus surface. This conformation is not recapitulated in the soluble rE protein. The overlap between the epitopes of 1F4 and another anti-DENV1 Ab 14c10, suggests that the DI-DII hinge region of the E protein is likely to be one of the principal epitopes in E protein that elicits a type-specific neutralizing Ab response. This work also suggests that the induction of neutralizing antibodies by vaccines incorporating soluble E protein monomers or dimers may be improved if the hinge angle of the E protein is identical to that on virus surface."

to:

"In summary, HMAb 1F4 binds to the DI and DI-II hinge regions of the E protein monomer and that Ab binding is likely to be sensitive to the hinge angle between DI-II. The overlap between the epitopes of 1F4 and another anti-DENV1 Ab 14c10, suggests that the DI-DII hinge region of the E protein is likely to be one of the principal epitopes in E protein that elicits a type-specific neutralizing Ab response. This work also suggests that the induction of neutralizing antibodies by vaccines incorporating soluble E protein monomers or dimers may be improved if the hinge angle of the E protein is identical to that on virus surface."

We removed Materials and Methods part sub-sections: ELISA binding assays and Non-reducing SDS-PAGE gel

We changed the Paper explained: Results section (Page 22) from:

"Human monoclonal antibody (HMAb) 1F4 was shown to be highly neutralizing *in vitro* and in an AG129 mouse model. We determined the structure of DENV1 complexed with Fab 1F4 to a resolution of 6 Å by using cryo-electron microscopy (cryo-EM). The structure showed that the antibody binds to domain (D) I, and the DI-DII hinge region on an envelope protein monomer. Interestingly, by ELISAs, we showed that this antibody specifically binds to E proteins when they were displayed on the virus surface but not to soluble recombinant E (rE) proteins or E proteins from lysed virus. Comparison of cryo-EM structures of virus E proteins to rE crystal structures showed that the E proteins on the virus had a conserved DI-DII hinge angle, whereas the hinge angle on the rE proteins is highly variable. As the DI-DII hinge forms part of the HMAb 1F4 epitope, we propose that HMAb 1F4 may be very sensitive to the conformation of this region. We also determined the mechanisms of neutralization of HMAb 1F4 in different cell lines. In Vero cells, the antibody prevents virus infection at a post-attachment step, whereas in DC-SIGN-expressing U937 cells, the HMAb can also prevent virus attachment. Using the cryoEM structure of 1F4 complexed with DENV, we discuss how the antibody could neutralize these steps of the virus infection."

to:

"Human monoclonal antibody (HMAb) 1F4 was shown to be highly neutralizing *in vitro* and in an AG129 mouse model. We determined the structure of DENV1 complexed with Fab 1F4 to a resolution of 6 Å by using cryo-electron microscopy (cryo-EM). The structure showed that the antibody binds to domain (D) I, and the DI-DII hinge region on an envelope protein monomer. Previous studies on HMAb 1F4 had demonstrated that it only binds to intact virus and not to recombinant envelope (rE) protein. Comparison of cryo-EM structures of virus E proteins to rE crystal structures showed that the E proteins on the virus had a conserved DI-DII hinge angle, whereas the hinge angle on the rE proteins is highly variable. As the DI-DII hinge forms part of the HMAb 1F4 epitope, we propose that HMAb 1F4 may be very sensitive to the conformation of this region. We also determined the mechanisms of neutralization of HMAb 1F4 in different cell lines. In Vero cells, the antibody prevents virus infection at a post-attachment step, whereas in DC-SIGN-expressing U937 cells, the HMAb can also prevent virus attachment. Using the cryoEM structure of 1F4 complexed with DENV, we discuss how the antibody could neutralize these steps of the virus infection."

We changed the Paper explained: Impact section (Page 22) from:

"We have identified and characterized an antibody that could potentially be used as a DENV1 therapeutic. The results also contribute significantly to vaccine design. Firstly, by comparing the HMAb 1F4-DENV1 structure to another potent HMAb 14c10-DENV1 structure, we observed an overlap at the DI-DIII hinge, suggesting that this region is likely to be one of the principal determinants in eliciting type-specific neutralizing antibodies in humans. The inclusion of this region is thus important for the development of an effective vaccine. In addition, we showed that HMAb 1F4, although binding to E protein monomer, is very sensitive to the E protein conformation and only recognizes the DI-DII hinge region in E as it is displayed on the virus surface. This conformation is held in proper configuration on the virus by the interactions between E ectodomains and also the E ectodomains with the E and M stem region. This has important implications for the development of an effective rE protein-based vaccine."

to:

"We have identified and characterized an antibody that could potentially be used as a DENV1 therapeutic. The results also contribute significantly to vaccine design. Firstly, by comparing the HMAb 1F4-DENV1 structure to another potent HMAb 14c10-DENV1 structure, we observed an overlap at the DI-DIII hinge, suggesting that this region is likely to be one of the principal determinants in eliciting type-specific neutralizing antibodies in humans. The inclusion of this region is thus important for the development of an effective vaccine. In addition, binding of HMAb 1F4 to E protein monomer is likely to be very sensitive to the hinge conformation. This conformation is held in proper configuration on the virus by the interactions between E ectodomains and also the E ectodomains with the E and M stem region. This has important implications for the development of an effective rE protein-based vaccine."

© EMBO
