# Supplementary material for: A potent anti-dengue human antibody preferentially recognizes the conformation of E protein monomers assembled on the virus surface
Source: EMBO Mol Med. 2014 Jan 14;6(3):358–71. doi: 10.1002/emmm.201303404 (PMC3958310; doi:10.1002/emmm.201303404)
Supplement: Supplementary file 2 [file emmm0006-0358-sd2.pdf]

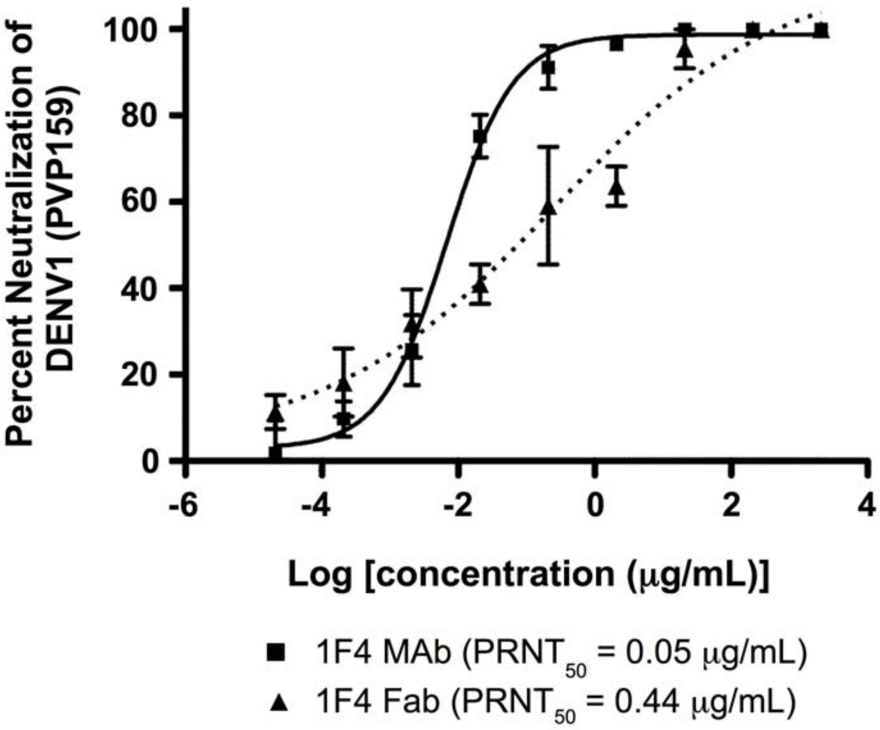


**Figure S1. Neutralization activity of HMAb 1F4 on DENV1 (PVP159).**

Both HMAb 1F4 and its Fab fragment neutralize DENV1 although the Fab fragment requires 10-fold higher concentration. This neutralization profile is similar to when DENV 1 strain West Pac was used (Fig 1). In this assay, the virus-antibody or Fab complex was added to BHK cells and the supernatant removed after 1 h incubation. RPMI/1% aquacide were layered onto the infected cells and it was then further incubated for 5 days at 37ºC before staining for plaques.
