# Supplementary figures and images for: A potent anti-dengue human antibody preferentially recognizes the conformation of E protein monomers assembled on the virus surface
Source: EMBO Mol Med. 2014 Jan 14;6(3):358–71. doi: 10.1002/emmm.201303404 (PMC3958310; doi:10.1002/emmm.201303404)

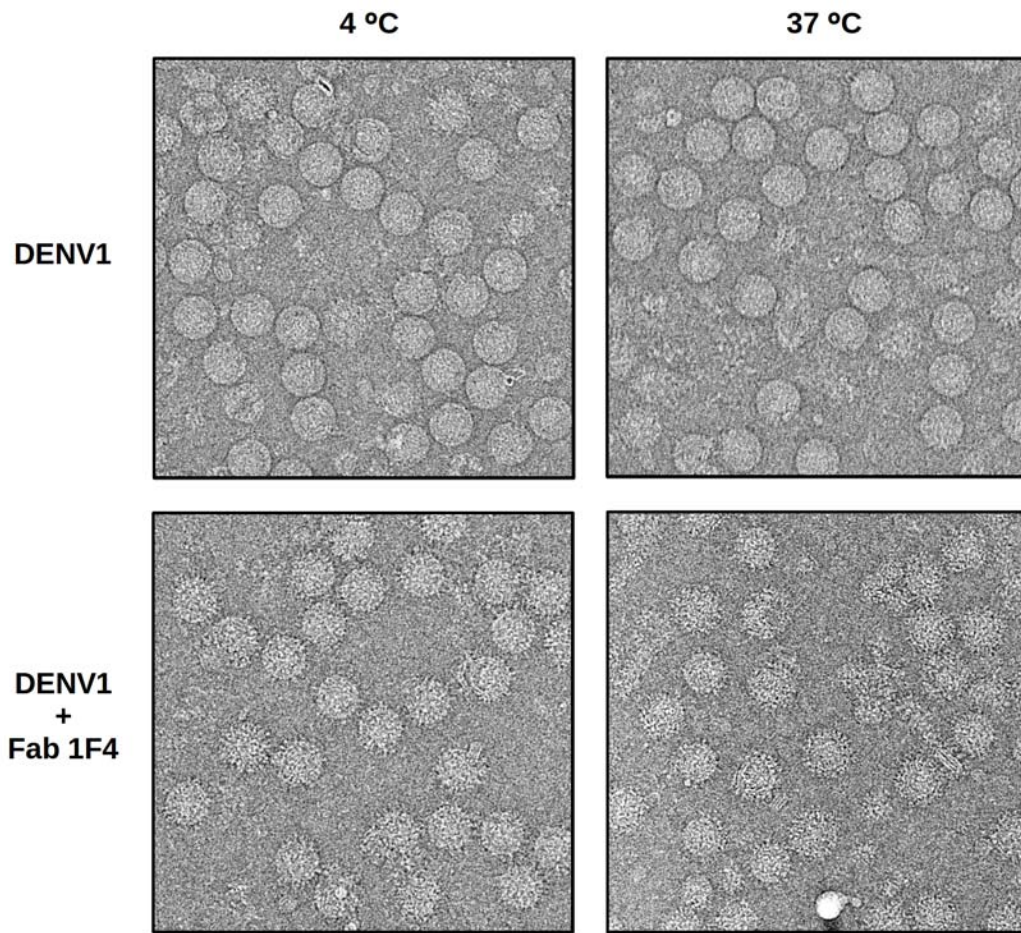

**Figure S2. Micrographs showing DENV1 controls and DENV1 complexed with Fab 1F4 at 4°C and 37°C.**

Supplement: Supplementary file 3 [file emmm0006-0358-sd3.pdf]
