# Supplementary material for: A potent anti-dengue human antibody preferentially recognizes the conformation of E protein monomers assembled on the virus surface
Source: EMBO Mol Med. 2014 Jan 14;6(3):358–71. doi: 10.1002/emmm.201303404 (PMC3958310; doi:10.1002/emmm.201303404)
Supplement: Supplementary file 4 [file emmm0006-0358-sd4.pdf]

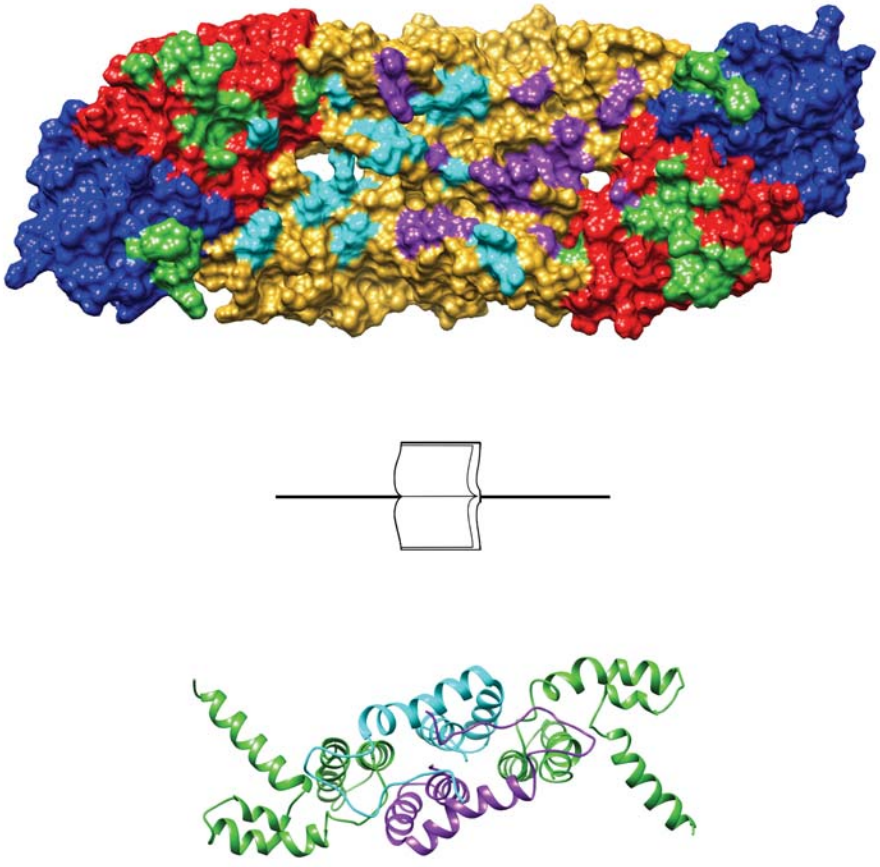

Figure S3. An open book representation of the interactions between the E ectodomain (top) with the stem region of E and M proteins (bottom) on DENV1 (Kostyuchenko et al, 2013)
