# Supplementary material for: A potent anti-dengue human antibody preferentially recognizes the conformation of E protein monomers assembled on the virus surface
Source: EMBO Mol Med. 2014 Jan 14;6(3):358–71. doi: 10.1002/emmm.201303404 (PMC3958310; doi:10.1002/emmm.201303404)
Supplement: Supplementary file 5 [file emmm0006-0358-sd5.pdf]

A potent anti-dengue human antibody preferentially recognizes the conformation of E protein monomers assembled on the virus surface

Guntur Fibriansah[1,2*], Joanne L. Tan[1,2*], Scott A. Smith[3,4], A. Ruklanthi de Alwis[5], Thiam-Seng Ng[1,2], Victor A. Kostyuchenko[1,2], Kristie D. Ibarra[6], Jiaqi Wang[1,2], Eva Harris[6], Aravinda de Silva[5], James E. Crowe, Jr.[4,7#] and Shee-Mei Lok[1,2#]

*- These authors contributed equally
#- co-corresponding authors

Addresses:
(1) Program in Emerging Infectious Diseases, Duke–NUS Graduate Medical School, Singapore.
(2) Centre for BioImaging Sciences, National University of Singapore, Singapore.
(3) Department of Medicine, Vanderbilt University, Nashville, TN.
(4) The Vanderbilt Vaccine Center, Vanderbilt University, Nashville, TN.
(5) Department of Microbiology and Immunology, University of North Carolina School of Medicine, Chapel Hill, NC.
(6) Division of Infectious Diseases and Vaccinology, School of Public Health, University of California, Berkeley, CA.
(7) Departments of Pediatrics and Pathology, Microbiology and Immunology, Vanderbilt University, Nashville, TN.

SUPPLEMENTARY INFORMATION – TABLE OF CONTENT

SUPPLEMENTARY FIGURES AND LEGENDS

Figure S1. Neutralization activity of HMAb 1F4 on DENV1 (PVP159). 2
Figure S2. Micrographs showing DENV1 controls and DENV1 complexed with Fab 1F4 at 4°C and 37°C. 3
Figure S3. An open book representation of the interactions between the E ectodomain (top) with the stem region of E and M proteins (bottom) on DENV1 (Kostyuchenko et al, 2013) 4
